# TFL Perforator Flap Complementing and Completing the ALT–AMT Flap Axis

**DOI:** 10.1055/a-2319-1564

**Published:** 2024-06-14

**Authors:** Dushyant Jaiswal, Bharat Saxena, Saumya Mathews, Mayur Mantri, Vineet Pilania, Ameya Bindu, Vinay Kant Shankhdhar, Prabha Yadav

**Affiliations:** 1 Department of Plastic and Reconstructive Surgery, Tata Memorial Hospital, Homi Bhabha National Institute, Mumbai, Maharashtra, India; 2 Department of Plastic and Reconstructive Surgery, Nanavati Max Super Speciality Hospital, Mumbai, Maharashtra, India; 3 Department of Plastic and Reconstructive Surgery, Sir H.N. Reliance Foundation Hospital and Research Centre, Mumbai, Maharashtra, India

**Keywords:** tensor fascia lata perforator flap, TFL perforator flap, ALT flap, chimeric ALT harvest, chimeric flaps

## Abstract

**Background** Anterolateral thigh (ALT) flap is the most common soft tissue flap used for microvascular reconstruction of head and neck. Its harvest is associated with some unpredictability due to variability in perforator characteristics, injury or unfavorable configuration for complex defects. Anteromedial thigh (AMT) flap is an option, but the low incidence and thickness restrict its utility. Tensor fascia lata (TFL) perforator (TFLP) flap is an excellent option to complement ALT. Its perforator is consistent, robust, in vicinity, and lends itself with the ALT perforator.

**Methods** This study was an analysis of 29 cases with a free flap for head neck reconstruction with an element of TFLP flap from July 2017 to May 2021.

**Results** All cases were primarily planned for an ALT reconstruction. There was absence of the ALT perforator in 16 cases but a sizable TFL perforator was available. In 13 cases, the complex defect warranted use of both ALT plus TFL in a conjoint (5), chimeric (5), and multiple (3) free flaps manner. Most common perforator location was septocutaneous between the TFL and gluteus medius. There was complete flap loss in two cases and partial necrosis in two. No adjuvant therapy was delayed.

**Conclusion** TFLP can reliably complement the ALT/AMT axis. Chimeric ALT–TFL can be harvested for large, complex, multicomponent, and multidimensional defects.

## Introduction

**Fig. 1:**
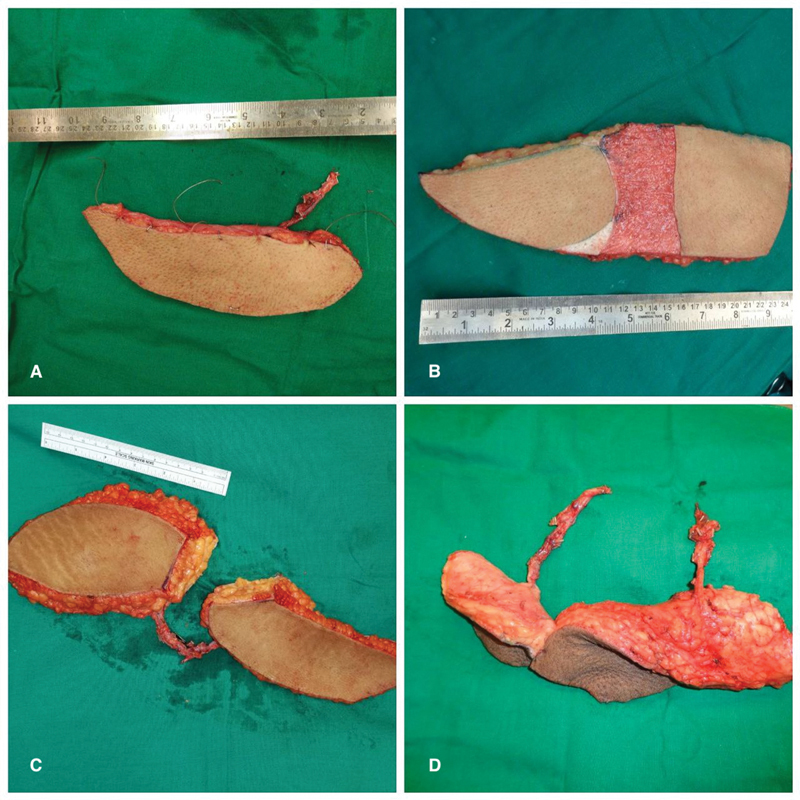
Various ALT TFL flap configuration. ( **A** ) TFL perforator. ( **B** ) Conjoint flap. ( **C** ) Chimeric flap. ( **D** ) Double free flap. ALT, anterolateral thigh; TFL, tensor fascia lata.

Anterolateral thigh (ALT) perforator flap has evolved to become a workhorse flap for Head and Neck (HN) reconstructions after cancer surgery. 1 Its harvest is still associated with an element of uncertainty due to variability in perforator characteristics; size, exact location in thigh, course through vastus lateralis muscle, and pedicle of origin. 2 3 4 This often translates into problems during harvest and partial or total skin island loss later. The anteromedial thigh (AMT) flap, in presence of a suitable perforator, can be used as an alternative or add on chimeric option. 5 The tensor fascia lata (TFL) perforator flap (TFLP) can fulfil all these roles of AMT flap with greater predictability and consistency. TFLP or lateral thigh flap with transverse incision is well-described for autologous breast reconstruction as an opportunistic choice in presence of thick lateral upper thigh, mostly as a secondary or rarely primary choice. 6 7 TFLP in HN reconstruction is scarce in literature. Considering the incidence of HN cancer in the Indian subcontinent, lesser body mass index (BMI) in these populations and routine use of thigh as a free flap donor site, understanding the versatility of TFL perforator is crucial and can be a force multiplier 8 ( Fig. 1 ). TFL perforator works synergistically with ALT for complex HN reconstruction. It yields certainty of flap harvest, robustness of vascularity, chimerism, and option to harvest multiple free flaps from the same thigh.

## Methods



**Supplementary Video 1** Video demonstration of chimeric antero-lateral thigh (ALT) flap and tensor fascia lata perforator (TFLP) flap for complex oromandibular defect. ALT, anterolateral thigh; BM, buccal mucosa; SCC, squamous cell carcinoma; TFL, tensor fascia lata.


Retrospective analysis of consecutive free flaps of HN reconstruction with a TFL perforator flap component ( *N* = 29), between July 2017 and May 2021, was done. Data were kept prospectively in MS-Excel, hospital electronic medical records, and personal logs of first author. All these patients were planned for a free ALT flap. Doppler marking was done along the vascular axis (anterior superior iliac spine (ASIS) to superolateral patella). A noncommittal incision is made 1.5 cm medial to this axis ( supplementary video 1 ). In all these patients, the incision was extended superiorly and posteriorly to look for the TFL perforator. Doppler signal was used in identifying the TFL perforator, which also guided the posterior extent of the incision.

## Results

During the study period, 884 ALT flaps were harvested for HN reconstruction of which 29 flaps (3.16%) were explored for a TFL component. Either due to the lack of an adequate ALT perforator or need for a chimeric configuration for reconstruction of complex defects.

**Fig. 2:**
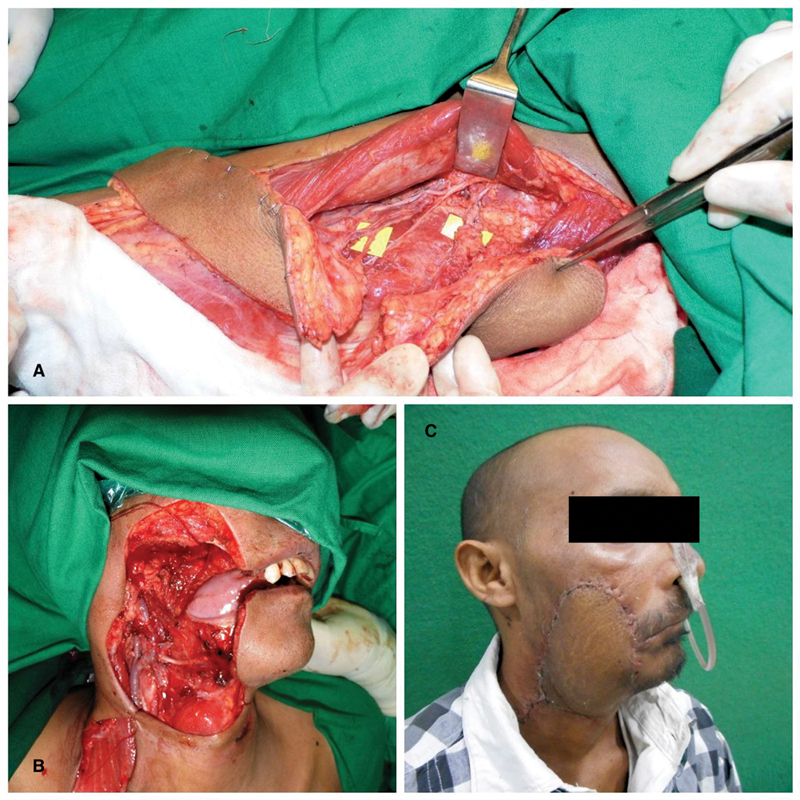
Right BM SCC (Defect: SM + upper alveolectomy + skin excision) reconstructed with Chimeric TFL and ALT flap. ( **A** ) In situ chimeric TFL and ALT flap. ( **B** ) Intraoperative defect. ( **C** ) Six-month postoperative result (postradiation). ALT, anterolateral thigh; BM, buccal mucosa; SCC, squamous cell carcinoma; TFL, tensor fascia lata.

**Fig. 3:**
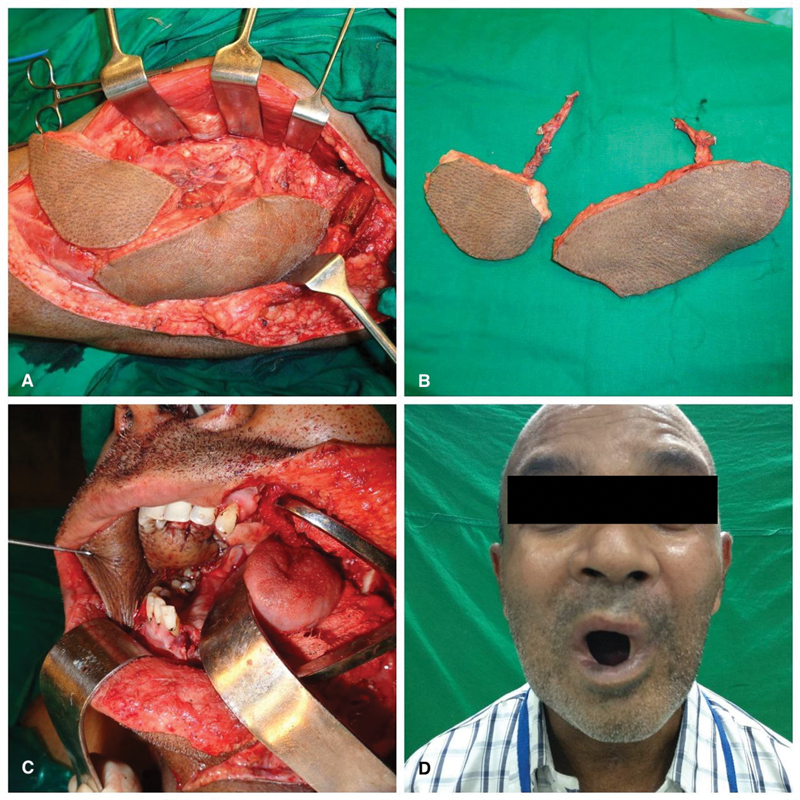
Bilateral BM SCC (Defect: B/L WLE of BM) reconstructed with double FF (free TFL and free ALT). ( **A** ) In situ flap divided on ALT and TFL perforator. ( **B** ) Divided free ALT and free TFL flap (double FF). ( **C** ) Intraoral defect with free ALT inset over right BM defect. ( **D** ) Six-month postoperative result (postradiation). ALT, anterolateral thigh; B/L, bilateral; BM, buccal mucosa; SCC, squamous cell carcinoma; TFL, tensor fascia lata; WLE, wide local excision.

TFL perforator was present in all 29 cases. In 16/29 cases, the ALT or AMT perforator was inadequate thus flap was harvested only on the TFL perforator. In rest of 13/29 cases, larger skin islands were needed or design requirement needed both ALT and TFL territories. Five of 13 of these flaps were harvested as conjoint flaps; 5/13 as chimeric where both the ALT and TFL pedicles joined each other enabling a single set of microvascular anastomoses (MVAs; Fig. 2 ). In 3/13 cases, the two pedicles were not joining or had multiple nerves entwined hence were harvested as two free flaps, needing two sets of MVAs ( Fig. 3 ). Table1 depicts design of flaps and average flap sizes ( Table 2 ).

**Table1:** Patient characteristics

Demographics	Results
Age	Average: 51.81 years (30–69)
Gender	Male: 20 Female: 9
Pathology	SCC oral cavity: 28 Clear cell carcinoma parotid: 1
Preop therapy	Chemotherapy: 7 Brachytherapy: 1 Radiotherapy: 1

Abbreviation: SCC, squamous cell carcinoma.

**Table 2:** Numbers and flap size in each category of tensor fascia lata flap

No.	TFLP flap type	Number	Maximum size	Average size	Mode of utilization
1.	TFLP flap	16	25 × 9 cm	16.75 × 7.93 cm	1. Mucosa only: 4 2. Skin cover only: 2 3. Skin cover and mucosal lining both a. De-epithelized inset: 9 b. Divided chimeric: 1
2.	Conjoint ALT + TFL	5	33 × 11 cm	23.14 × 8.42 cm	1. ALT a. Skin cover: 3 b. Mucosal lining: 2 2. TFL a. Skin cover: 2 b. Mucosal lining: 3
3.	Chimeric ALT + TFL	5	26 × 8 cm	22 × 7.5 cm	1. ALT a. Skin cover: 4 b. Mucosal lining: 1 2. TFL a. Skin cover: 1 b. Mucosal lining: 4
4.	Double free flap (ALT and TFL flaps from the same thigh)	3	ALT: 25 × 12 cm TFL: 13 × 7 cm	ALT: 15.33 × 9.5 cm TFL: 10.75 × 8.6 cm	1. ALT a. Skin cover: 0 b. Mucosal lining: 3 2. TFL a. Skin cover: 2 b. Mucosal lining: 1

Abbreviations: ALT, anterolateral thigh; TFL, tensor fascia lata; TFLP, tensor fascia lata perforator.

The most common course of the perforator was septocutaneous (between the TFL and gluteus medius; 27/29) followed by a musculocutaneous course (through the TFL muscle; 2/29), and no (0/29) case had a septocutaneous course between rectus femoris and TFL. Seven of 29 cases had multiple TFL perforators. Of these seven cases, six had two septocutaneous perforators and one had multiple musculocutaneous perforator. The length of the pedicle was sufficient for primary tension-free anastomosis in all cases, no vein grafts were needed. Average pedicle length was 7 cm. All 19/29 patients planned, received postoperative radiation therapy on scheduled time.

Twenty-seven of the 29 flaps survived. Out of the two flaps lost (7.14%), one had venous insufficiency due to venous thrombosis and was replaced with a scalp flap. One patient, with past history of radiation, had arterial insufficiency and acute bleeding due to anastomosis dehiscence. This lost flap was replaced by a pedicled latissimus dorsi flap. Two flaps had marginal necrosis requiring secondary suturing only. There were no flap complications as a result of radiation therapy.

Donor site complication was present in four cases (13.79%). Donor sites were closed primarily for 17/29 patients of which 1 required resuturing and 2 required secondary skin grafting. The average width in which the flap was closed primarily was 7.29 cm and the maximum was 12 cm. Skin grafting was done in 12/29 patients, of which 1 patient had partial graft loss which was managed conservatively ( Table 3 ).

**Table 3:** Master chart of all participating patients in the study

Serial no.	Age	Primary diagnosis	Resection Sx	Configuration	Flap size (cm × cm)	Number and course of TFL Perforators	Flap usage	Donor closure	Flap complications
1	54/M	Rt BM SCC	Rt SM + Upper alveolectomy + Total parotidectomy + ITF clearance + Skin	Double FF	ALT: 25 × 12; TFL: 10 × 12	Multiple (MC)	TFL: Skin cover, ALT: Mucosal lining	PC	None
2	30/M	Rt BM SCC	Rt SM + Upper alveolectomy + Skin	Double FF	ALT: 9 × 7, TFL: 8 × 7	1 (SC)	TFL: Skin cover + filler, ALT: Mucosal lining	PC	Gaping of donor wound, secondarily grafted
3	51/F	Rt BM SCC	Rt SM + Upper alveolectomy + Skin	TFLP flap	25 × 9 (flap thinned)	2 (SC)	Skin and mucosal lining with de-epi	STSG	None
4	65/F	Recc Rt BM SCC (previously operated FRAFF)	Lt SM + Skin	TFLP flap	15 × 7	1 (SC)	Skin and mucosal lining with de-epi and filler	PC	Re-suturing of donor wound
5	59/M	Lt BM SCC	Lt WLE + MM + Upper Lip 50%	TFLP flap	23 × 12 (Flap Thinned)	2 (SC)	Skin and mucosal lining with de-epi	STSG	Flap marginal necrosis requiring Secondary Suturing
6	36/M	Lt BM SCC	Lt Hemi mandibulectomy + Skin	Conjoint flap	24 × 10	2 (SC)	TFL: Skin cover, ALT: Mucosal lining with de-epi	STSG	None
7	61/M	Lt BM SCC	Lt SM + Upper alveolectomy	TFLP flap	14 × 7	1 (SC)	Mucosal lining and filler	PC	None
8	49/M	Rt BM SCC	Rt Hemimandibulectomy + Upper alveolectomy + Skin	Conjoint flap	28 × 8	1 (SC)	TFL: Skin cover, ALT: Mucosal lining with de-epi	STSG	None
9	67/F	Lt BM SCC	Lt WLE + MM + Upper alveolectomy + Skin	Conjoint flap	20 × 8 (flap thinned)	1 (SC)	TFL: Skin cover, ALT: Mucosal lining with de-epi	PC	None
10	69/F	Rt BM SCC	Rt SM	TFLP flap	10 × 7	2 (SC)	Mucosal lining and filler	PC	None
11	42/F	Rt BM SCC	Rt WLE + Upper alveolectomy + Palatal excision	TFLP flap	24 × 7	1 (SC)	Mucosal lining and filler	PC	None
12	52/M	B/L BM SCC	B/L WLE	Double FF	ALT: 11 × 7, TFL: 13 × 7	1 (SC)	TFL: Left BM, ALT: Rt BM	PC	None
13	57/M	Rt BM SCC	Rt WLE + MM	TFLP flap	8 × 6	1 (SC)	Mucosal lining	PC	None
14	48/M	Recc Lower alveolus SCC	Mid SM + Upper alveolectomy + Skin	TFLP flap	19 × 11	2 (SC)	Skin cover	STSG	Total Flap loss due to Venous Insufficiency
15	59/M	Clear cell Ca Parotid	Total parotidectomy	TFLP flap	10 × 7	1 (SC)	Skin cover and filler	STSG	None
16	44/M	Lt BM SCC	Lt SM + Upper alveolectomy + Skin	Conjoint flap	20 × 8	1 (SC)	Skin and mucosal lining with de-epi	STSG	None
17	43/M	Lt BM SCC	Lt SM + Upper alveolectomy + Skin	Chimeric flap	TFL: 10 × 8, ALT: 14 × 8	1 (SC)	TFL: Mucosal lining, ALT: Skin cover	STSG	None
18	47/F	Rt BM SCC	Rt SM + Upper alveolectomy + Skin	Conjoint flap	21 × 7	2 (SC)	Skin and mucosal lining with de-epi	PC	None
19	52/M	Rt BM SCC	Rt SM + Upper alveolectomy + Hemi glossectomy + Skin	Chimeric flap	TFL: 8 × 7, ALT: 10 × 8	1 (SC)	TFL: Skin cover, ALT: Mucosal lining and tongue	STSG	Total flap loss due to arterial insufficiency
20	48/M	Lt BM SCC	Lt SM + Upper alveolectomy + Maxillectomy + ITF clearance + Skin	TFLP flap	27 × 7	1 (SC)	Skin and mucosal lining with de-epi and chimeric TFL muscle for maxillary defect	STSG	Flap marginal necrosis requiring Secondary Suturing
21	43/M	Lt BM SCC	Lt WLE + MM + Skin	TFLP flap	12 × 6	1 (SC)	Skin and mucosal lining with de-epi	PC	Donor re-grafting
22	34/M	Tongue SCC	Near total glossectomy + MM	TFLP flap	12 × 8	1 (SC)	Mucosal lining	PC	None
23	69/F	Rt BM SCC	Lt Hemi mandibulectomy + Upper alveolectomy + Maxillectomy	TFLP flap	24 × 8	1 (SC)	Mucosal lining	PC	None
24	66/F	Recc Lt BM SCC (Previous operated PMMC)	left BM WLE + Palatal excision + Skin	TFLP flap	16 × 8	1 (SC)	Skin and mucosal lining with de-epi	PC	None
25	43/M	Lt BM SCC	Lt SM + Upper alveolectomy + Skin + Maxillectomy	Chimeric flap	TFL: 17 × 8; ALT: 16 × 11 + vascularized iliac bone graft 4 × 3.5	1 (SC)	TFL: Mucosal lining, ALT: Skin cover	STSG	None
26	68/M	Recc Rt BM SCC (previous operated FFOCF)	Right SM + Upper alveolectomy + Skin	Chimeric flap	TFL: 16 × 8, ALT: 13 × 11	1 (SC)	TFL: Mucosal lining, ALT: Skin cover	STSG	Donor re-grafting
27	55/M	Recc Lower lip SCC	Complete Lower lip excision and MM	TFLP flap	16 × 4	1 (SC)	Skin cover and mucosal lining	PC	None
28	66/F	Left lower alveolus SCC	Lt SM + Skin	TFLP flap	13 × 8	1 (SC)	Skin and Mucosal lining with de-epi	PC	None
29	32/M	Lt BM SCC	Lt SM + Upper alveolectomy + Maxillectomy + ITF clearance	Chimeric	TFL: 12 × 7, ALT: 13 × 7	1 (SC)	TFL: Mucosal lining, ALT: Skin cover	PC	Flap secondary suturing

Abbreviations: ALT, anterolateral thigh; B/L, bilateral; BM, buccal mucosa; Ca, carcinoma; F, female; FFOCF, free fibula osteocutaneous flap; FRAFF, free radial artery forearm flap; ITF, infratemporal fossa; Lt, left; M, male; MC, musculocutaneous; MM, marginal mandibulectomy; PC, primary closure; PMMC, pectoralis major myocutaneous flap; Rec, recurrent; Rt, right; SC, septocutaneous (between TFL and gluteus medius); SCC, squamous cell carcinoma; SM, segmental mandibulectomy; STSG, split thickness skin graft; TFL, tensor fascia lata; TFLP, tensor fascia lata perforator; WLE, wide local excision.

## Discussion

The popularity of ALT flap is attributed to donor site with abundant skin and soft tissue usually amenable to primary closure; generally robust perforators; long pedicle length and sizeable lumen. The possibility of having an unfavorable perforator anatomy cannot be ruled out. Incidence as high as 5.4% has been reported. In such scenario the likelihood of finding a good perforator on the contralateral thigh is also unlikely. 2 9 The AMT flap, in presence of a suitable perforator, can be preferred over ALT or be used as salvage option in case of injury or in a complementary role as chimeric or additional free flap. Its low incidence restricts it in this role. 5

**Fig. 4:**
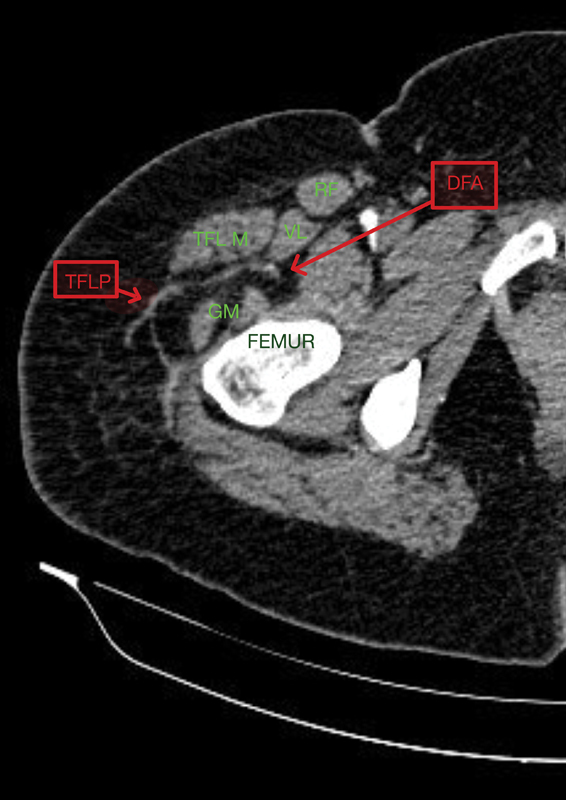
CT angiogram image of TFL perforator (TFLP) originating from the deep femoral artery (DFA) and emerging between the TFL muscle and gluteus medius (GM) muscle. RF, rectus femoris; TFL, tensor fascia lata; VL, vastus lateralis.

**Fig. 5:**
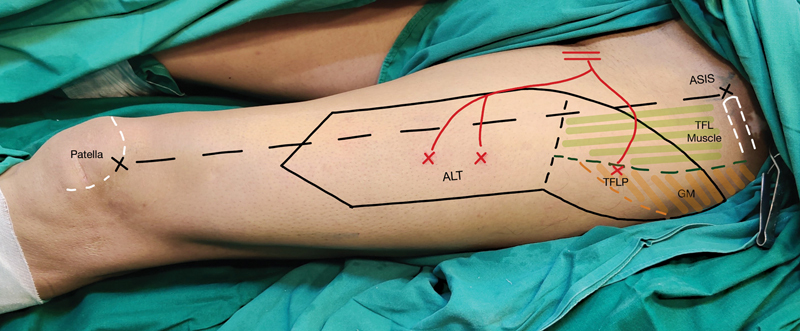
Surface marking of the ALT and TFL flap territory and perforator location. A noncommittal incision which is 1.5 cm medial to the vascular axis of the ALT flap (anterior superior iliac spine and the superolateral patella). To approach the TFL perforator, the same incision is extended posterosuperiorly guided by the handheld Doppler signal. ALT, anterolateral thigh; TFL, tensor fascia lata; TFLP, tensor fascia lata perforator.

TFLP can fulfil all these roles of AMT. Clinical and MRI/CT imaging studies show greater consistency in presence of TFL perforator as c.f. AMT 10 11 12 13 ( Fig. 4 ). In our high-volume and resource-constrained setup, preoperative imaging has cost and logistic compulsions, hence it is not used routinely. A noncommittal, freestyle perforator to pedicle-based approach is advisable before committing to a flap incision and design. 5 The proximal and posterior extension of the straight-line noncommittal incision can give a good exposure to the TFL perforator, guided with the handheld Doppler signal ( Fig. 5 ). It is generally located 7 to 10 cm below ASIS and 4 to 6 cm behind the vascular axis of the ALT flap. A complementary relationship exists between perforators in the ALT, AMT, and TFL territory supplied by different tributaries of LFCA.

TFLP (as lateral thigh flap) is well-described for breast reconstruction as a rare primary or secondary choice. 14 Few case reports and series exist for TFLP use in HN reconstruction. 1 11 15 However, its larger application as a lifeboat or in complex HN defects has not been described in literature.

TFL muscle in the proximal thigh is enclosed within the ventral and dorsal layers of deep fascia. The possible perforators to the skin are:

1. Dorsal/Posterior septocutaneous perforator/s: generally one or two, large in size, travelling between the TFL and gluteus medius muscles. These are the most robust and consistent perforator as shown in our series as well.
2. Musculocutaneous perforators: generally one to three in number, small to medium size are present, traversing the substance of TFL muscle.
3. Ventral/Anterior septocutaneous perforators: These are rare and travel in the “ALT septum” or adherent to the ventral layer of deep fascia. Often mistaken for ALT or “Oblique” branch perforators. 5

The perforator is traced through this septum to the main pedicle (transverse branch of lateral circumflex femoral artery) yielding a pedicle length between 6 and 9 cm usually sufficient for HN reconstruction.

TFL perforator flap was preferred over a TFL musculocutaneous flap in our series. Visible perforators guarantee inclusion of the vascular basis in the flap, allowing small flaps (8 × 6 smallest in our series) and permits surgical thinning for superior contouring liberty and possibility of chimeric flaps based on same TFL pedicle (one case in our series). Flap is thinner with longer pedicle length as muscle is excluded hence better suited for HN reconstruction.

Flaps with a TFL perforator flap component were harvested in the following situations and morphology.

1. **TFL perforator flap** : A single skin island flap, small or moderate in size, was harvested when ALT/AMT perforator were unavailable, unsatisfactory, or injured.
2. **Conjoint ALT–TFL perforator flap** : In some cases, a very large single skin island flap was needed, and the ALT perforators seem to be insufficient to sustain the whole flap. In these cases, a robust TFL perforator flap component can be added to the flap. Hence, a larger skin flap can be harvested, encompassing the TFL and ALT territory while preserving their individual vascular supply. Recruitment of TFL would avoid marginal flap necrosis, especially when parts of skin are de-epithelized and turned for inset and contouring.
3. **ALT–TFL chimeric perforator flap** : It was used when large, multicomponent, multiaxial defect needing two skin islands were needed. ALT–TFL chimeric flap was harvested if pedicles were joining each other and it was possible to retrieve the flap without significant nerve damage or need to ligate too many branches. In case of intervening nerves “Divide and Deliver technique” was used. 16 Now we prefer ALT–TFL combination over ALT–AMT, as it allows primary closure of donor.
4. **ALT and TFL perforator flaps** : In the above situation if the pedicles are not joining, joining but retrieval would entail significant nerve damage or too many branches to muscles, especially rectus femoris need to be ligated, two separate free flaps were harvested from the same donor site ( Fig. 1 ).

The expendability of TFL muscle and donor site morbidity needs consideration. TFL is a Type 1 muscle, hence harvesting the pedicle might render the muscle ischemic if not necrotic. 17 If there was any doubt of perfusion, the muscle or a part of it was debrided. Occasionally, contrary to expectation as TFL is Mathes and Nahai Type 1 muscle, if the muscle was well-perfused it was retained. Occasionally, muscle might survive from proximal branches from superficial femoral vessels. The TFL muscle can be harvested as a chimeric component and be used for filling/sealing the maxilla or nasal cavity. The iliac crest bone, around 4 cm anterior bone outer lip, can also be harvested with the TFL muscle. TFL expendability is subject to debate. It only has a supportive role in hip and knee movement and stability and is not a prime mover for any movement. Donor morbidity from sacrificing the TFL muscle can have effects over the gait initially. There are not much objective data on long-term functional outcomes. The argument stands that even with harvesting a large ALT (as an alternative to ALT + TFL flap), a significant deep fascia defect renders the muscle functionless as it loses its insertion.

The donor site, being over greater trochanter, should be preferably closed primarily without tension. Closures under tension tend to breakdown and may eventually require skin grafting over an unfavorable bed and poor cosmesis.

Incidence of complete flap loss in our study was 7% (2/29). This can be attributed to previous radiotherapy (arterial anastomotic dehiscence) and microsurgical technical error (venous anastomotic thrombus) rather than the nature of the flap. Marginal necrosis in two patients could be due to the TFL perforator insufficiency in a large flap with average length of 25 cm. However, the incidence was low (7%) and was corrected promptly with secondary suturing without any delay in adjuvant therapy ( supplementary material available online).

TFLP territory is conventionally thicker than the ALT territory and is our primary choice over ALT/AMT when small but thick flap is desired, especially in low BMI patients. The consistent presence, size, and course of a septocutaneous perforator between the TFL and gluteus medius muscles makes the TFL flap is an excellent backup and also allows for a definite ability to harvest a chimeric flap from the same donor site.

### Conclusion

TFL perforator is consistent and robust. We recommend harvesting flaps from the thigh with a noncommittal straight-line incision initially, perceiving ALT–AMT–TFL perforators as a complementary unit. TFLP can be used to counter ALT/AMT unavailability, injury, suboptimal quality or need of a thicker flap. Chimeric ALT–TFL can be harvested for large, complex, multicomponent, and multidimensional defects.
