# Supplementary material for: TFL Perforator Flap Complementing and Completing the ALT–AMT Flap Axis
Source: Arch Plast Surg. 2024 Jun 14;51(4):408–16. doi: 10.1055/a-2319-1564 (PMC11257743; doi:10.1055/a-2319-1564)
Supplement: Supplementary file 2 — Supplementary Material [file 10-1055-a-2319-1564-s23aug0431oa.pdf]

TFL perforator flap – complementing and completing the ALT-AMT flap axis

ALT flap

- ALT is a workhorse flap for head neck reconstruction

- Popularity of ALT flap
  - Abundant skin and soft tissue
  - Robust blood supply
  - Good pedicle length and lumen size

Is ALT fool-proof ?

- Uncertainty with respect to the number, size, site and course of perforators.

- Problems with ALT
  - Unfavorable perforator anatomy (5.4%)

- Imaging
  - Hand held doppler – limited accuracy, operator dependent
  - CT / MRI – Although reliable (Cost and logistics issue)

LCFA Axis

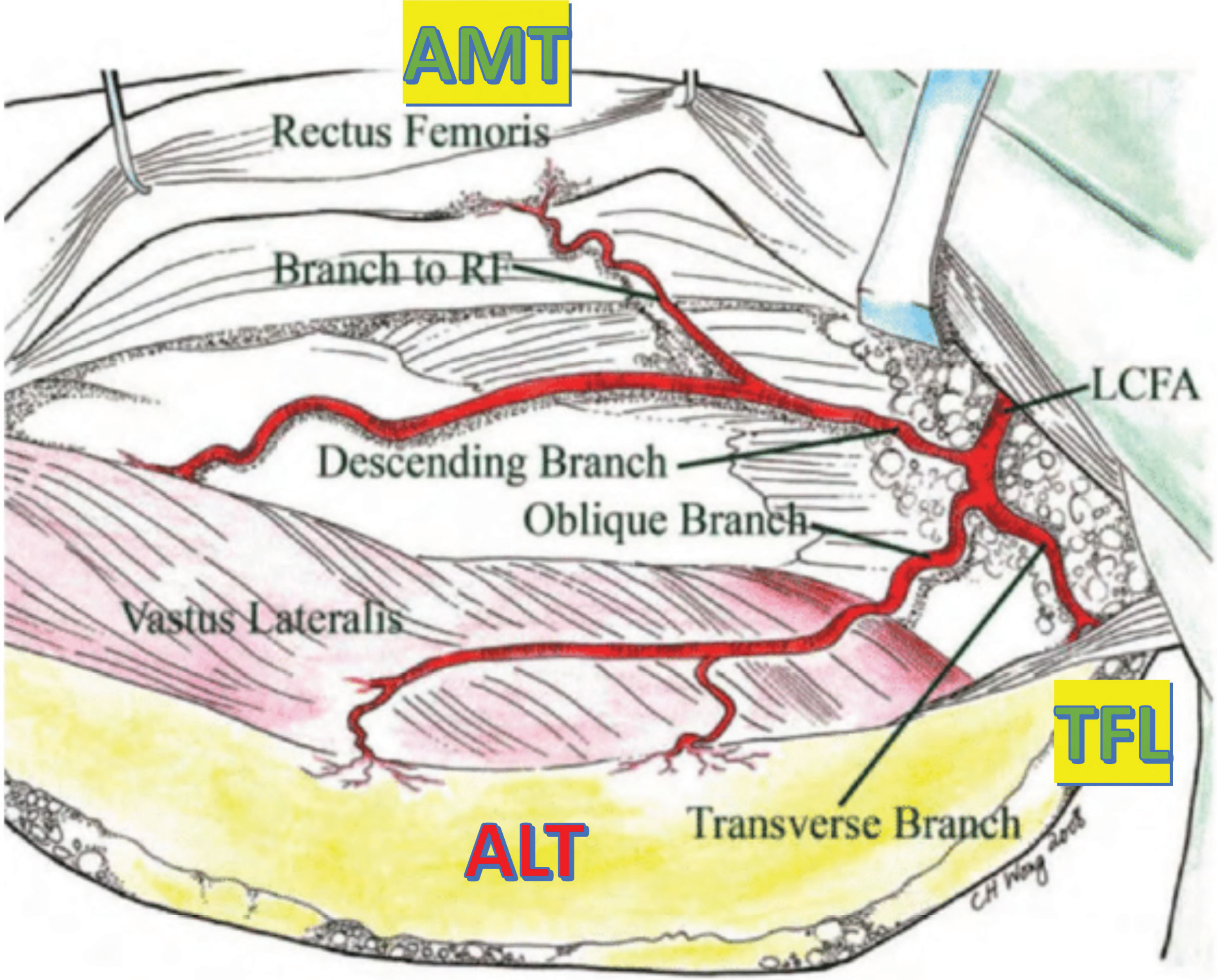
AMT
Rectus Femoris
Branch to RF
LCFA
Descending Branch
Oblique Branch
Vastus Lateralis
TFL
ALT
Transverse Branch
CH Wang 2018

CT image

TFL perforator originating from the Deep Femoral Artery (DFA) between the Tensor Fascia Lata (TFL) & Gluteus Medius Muscle (GM)

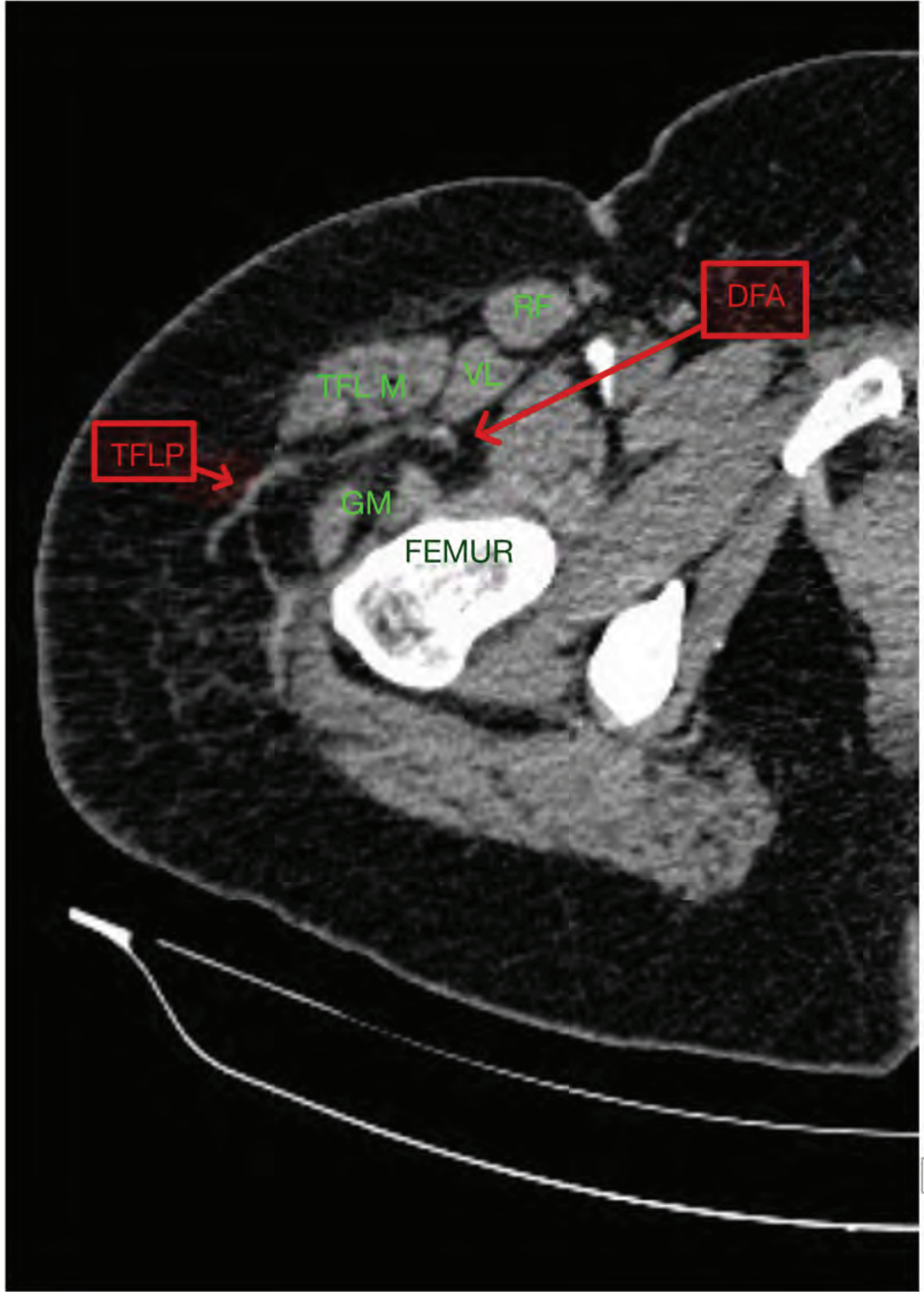

TFL perforator

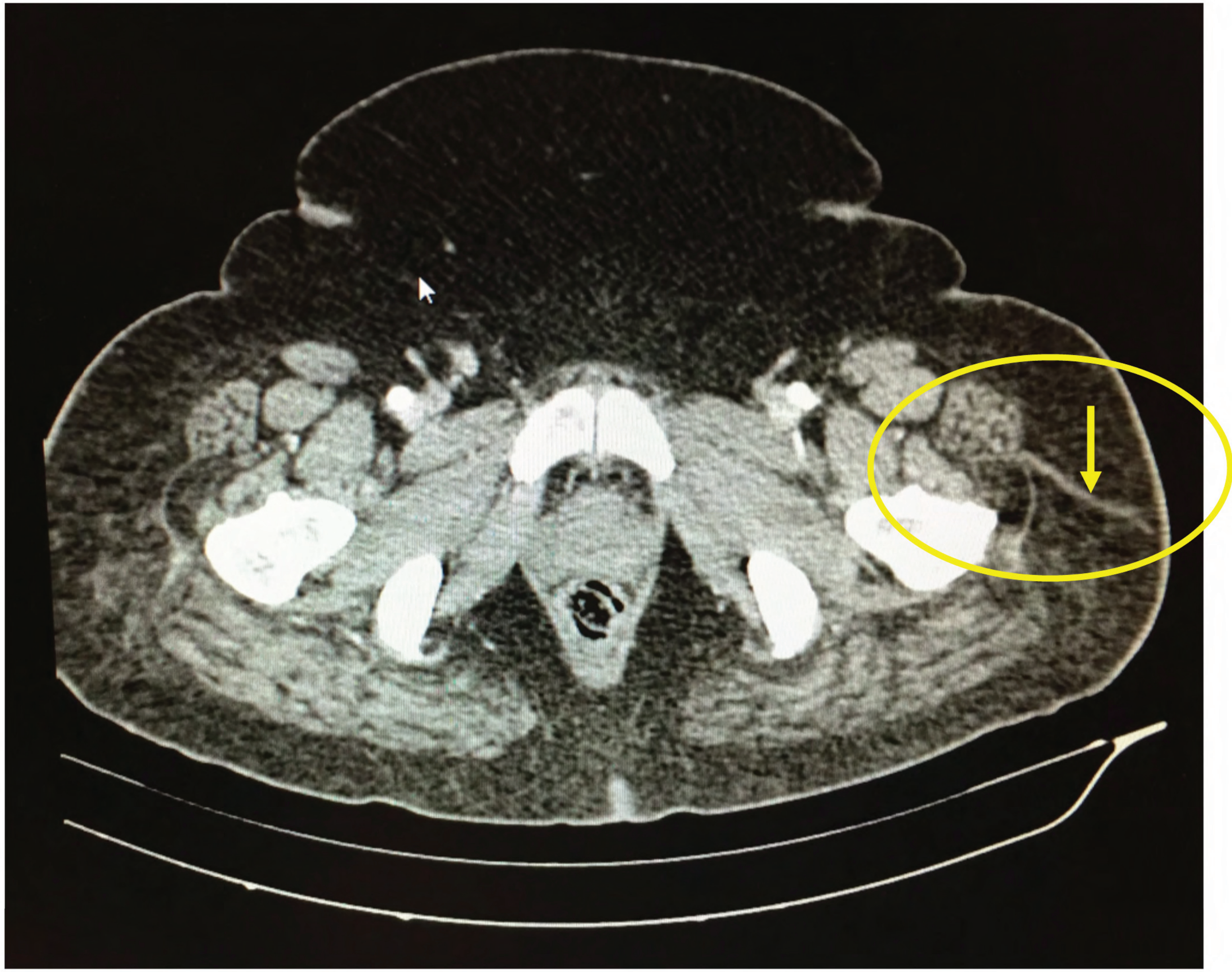

Material and Methods

- Retrospective analysis of free flaps with a TFL component
- *N* = 29
- July 2017 to May 2021
- Data - MS-EXCEL, hospital EMR and personal logs of the author

Noncommittal approach

- All these patients were planned for a free ALT flap
- A non-committal incision is taken 1.5 cm medial to the vascular axis of ALT
- In all these patients the incision was extended superiorly and posteriorly to look for the TFL perforator

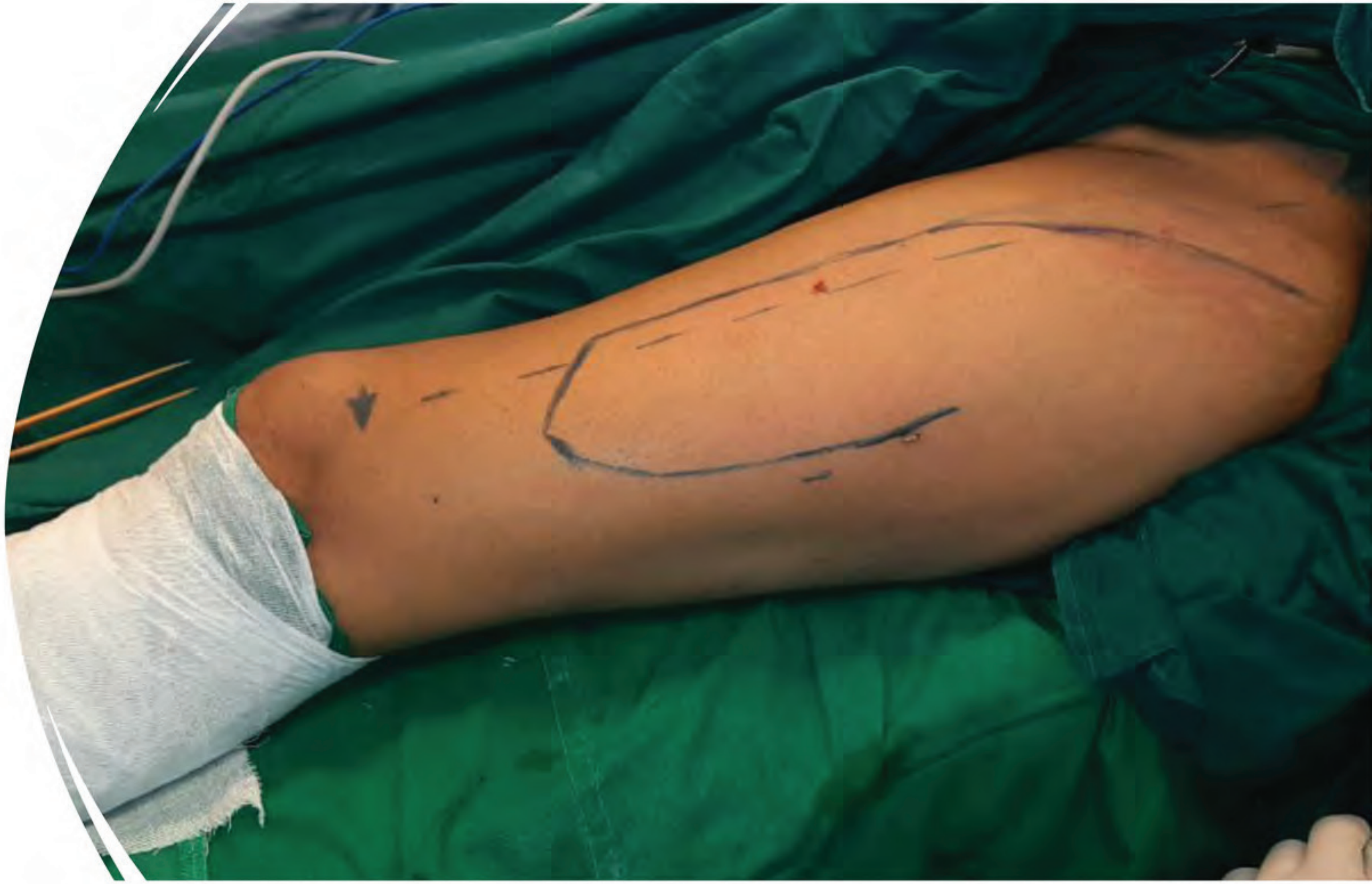

Flap Configurations

A – TFL perforator
B – Conjoint flap
C – Chimeric flap
D – Double free flap

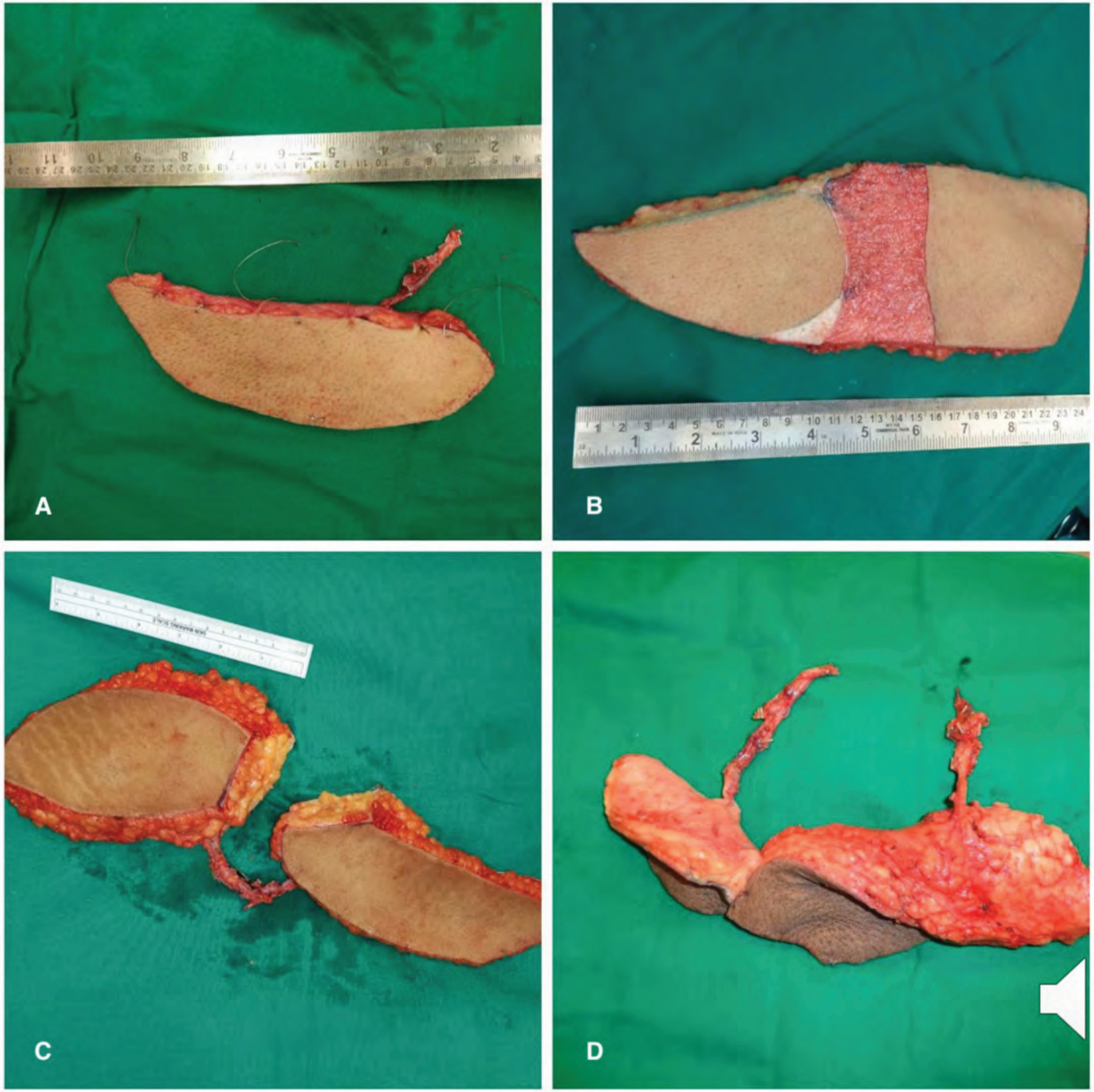

Result

Demographics	Results
**Age**	Average – 51.81 years (30-69)
**Gender**	Male -20<br>Female -9
**Pathology**	SCC oral cavity – 28<br>Clear cell carcinoma parotid -1
**Preop therapy**	Chemotherapy - 7<br>Brachytherapy-1<br>Radiotherapy -1

Results

Total $N = 29$

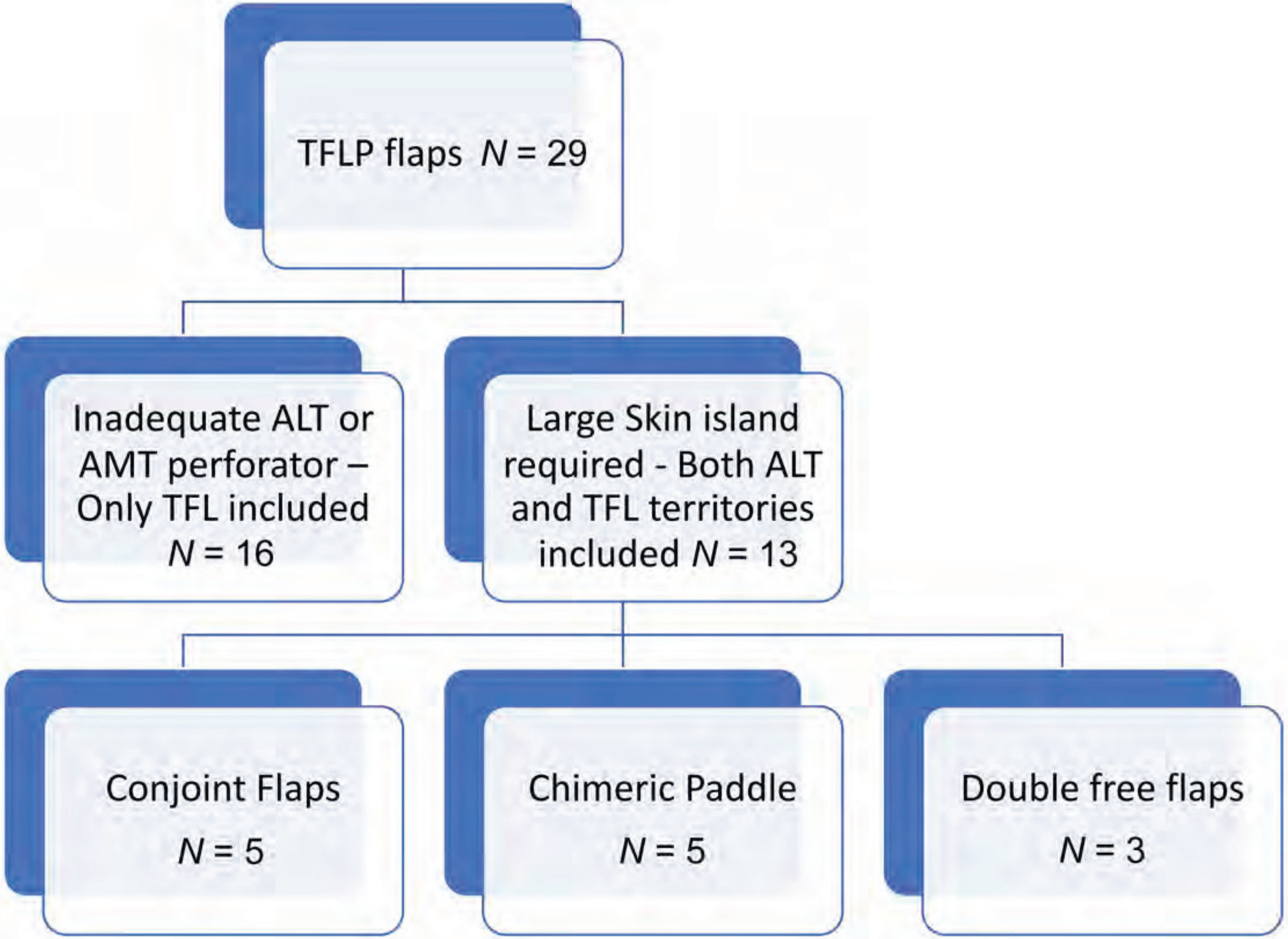

Results

- Course of perforator :

Septo-cutaneous (TFL and GM)	Musculocutaneous	Septocutaneous (TFL and RF)
27/29	2/29	0/29

- Pedicle length - Primary tension free anastomosis in all cases (no vein grafts).

- All 19/29 patients planned received post-operative radiation therapy on scheduled time

Donor Complications

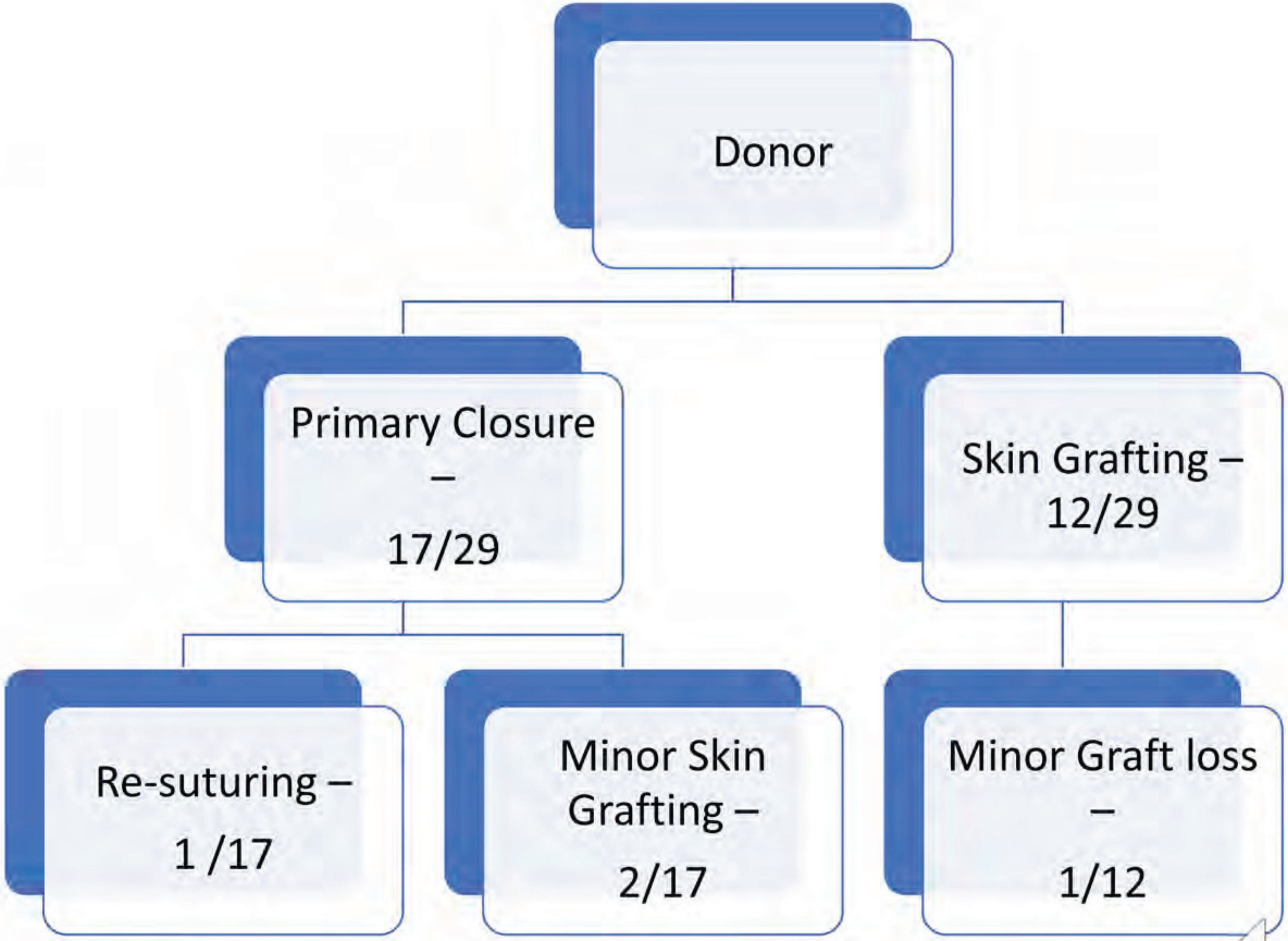

Flap Complications

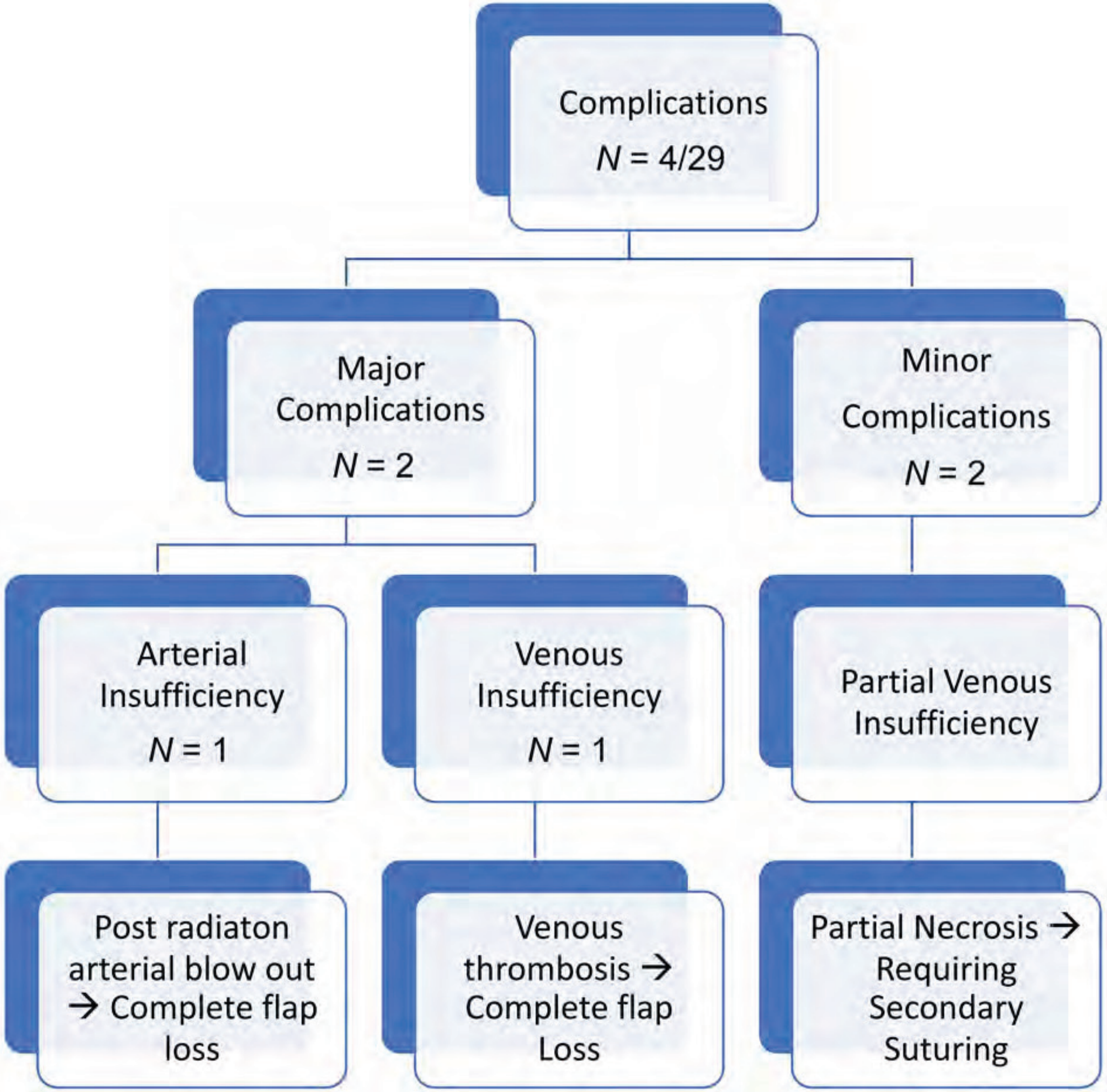

Pure TFLP flap - Single paddle

A – Left BM SCC defect with WLE and Marginal Mandibulectomy
B – TFLP flap
C – Intraoperative flap Inset
D – 1-year Postoperative follow-up (Postradiation)

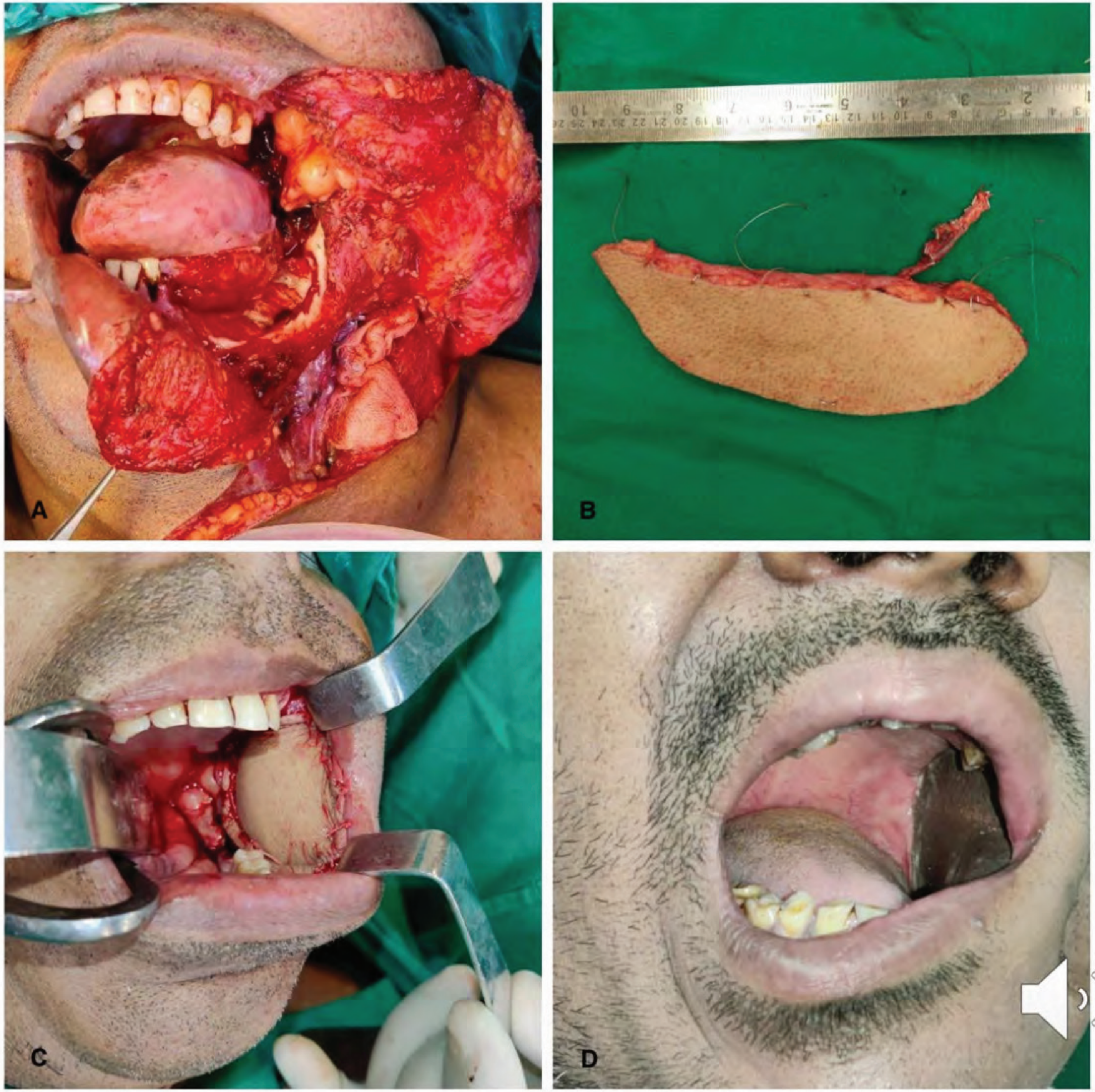

Conjoint Flap – TFL and ALT

A – Left BM SCC defect with Composite resection
B – De-epithelized TFL & ALT flap
C – Intraoperative flap Inset
D – 8 months Postoperative follow-up (Postradiation)

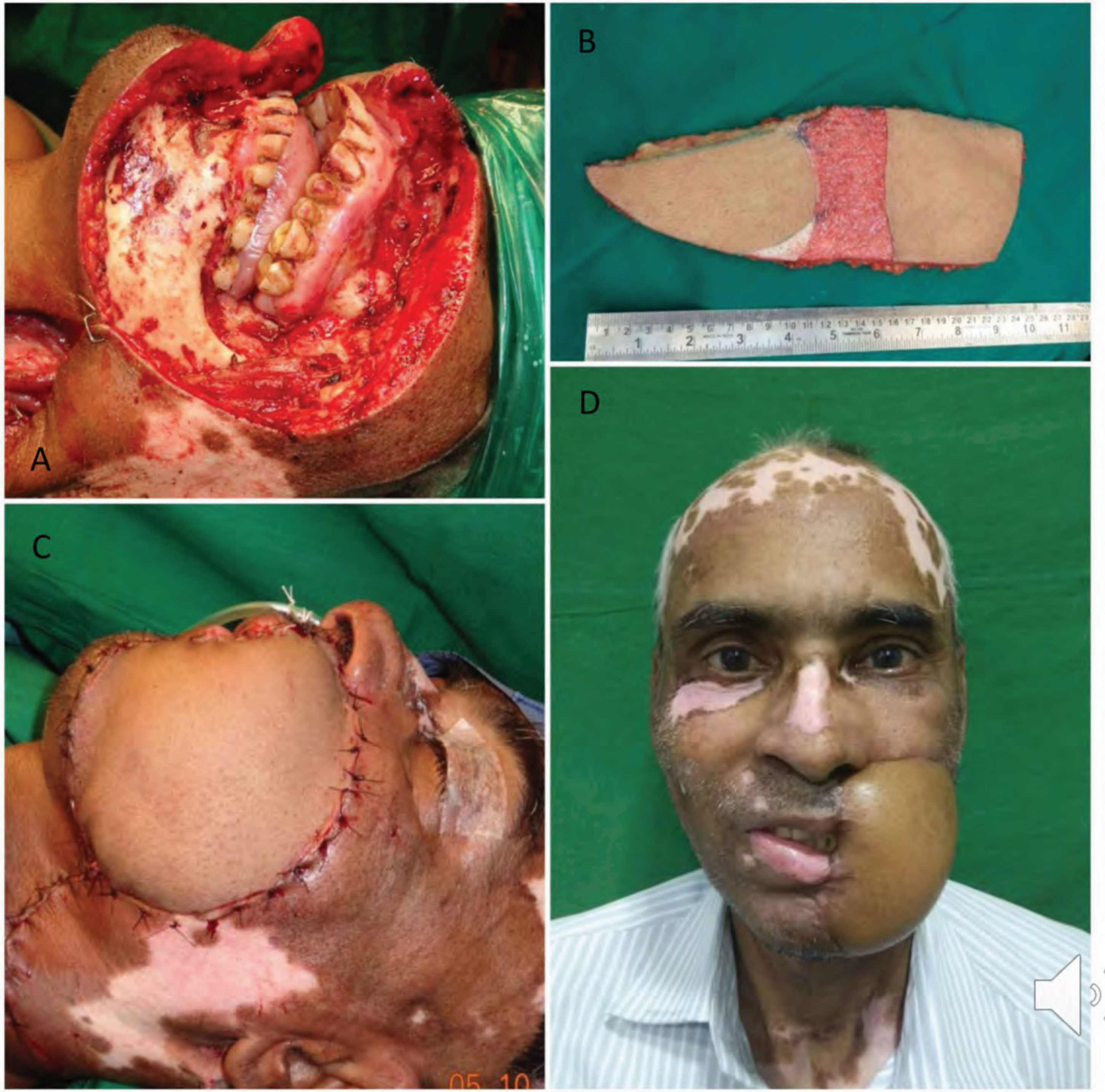

Chimeric Flap – TFL and ALT

A –Divided Chimeric flap (TFL & ALT)
B – Segmental Mandibulectomy + Upper alveolectomy + Skin defect
C – 1-year Postoperative follow-up (Postradiation)

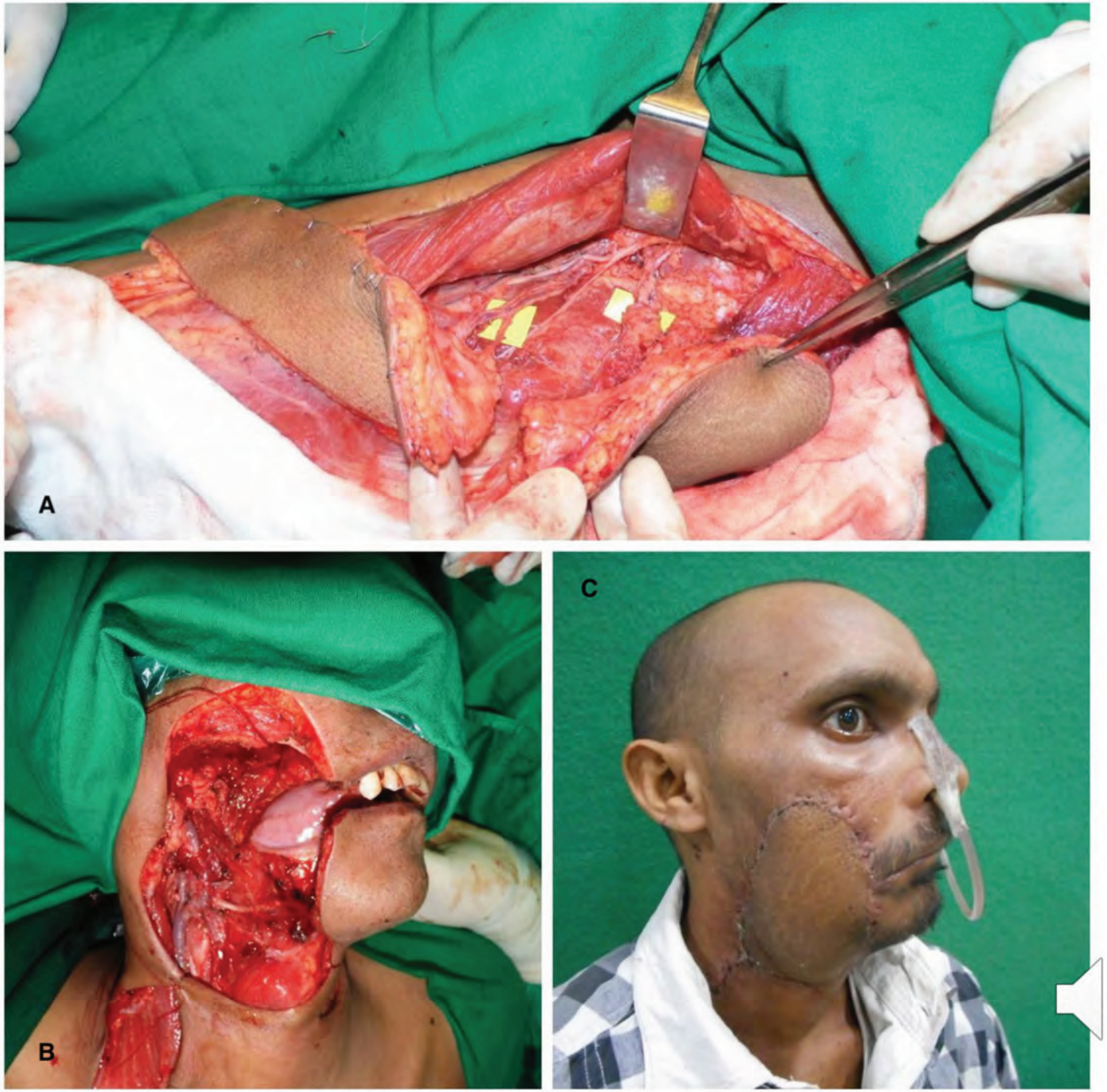

Double FF – TFL and ALT

A – In-situ flap divided on ALT and TFL Perforator
B – Divided Free ALT and Free TFL flap (Double FF)
C – Intra oral defect with Free ALT inset over right BM defect
D – 6-month postoperative figure (Postradiation)

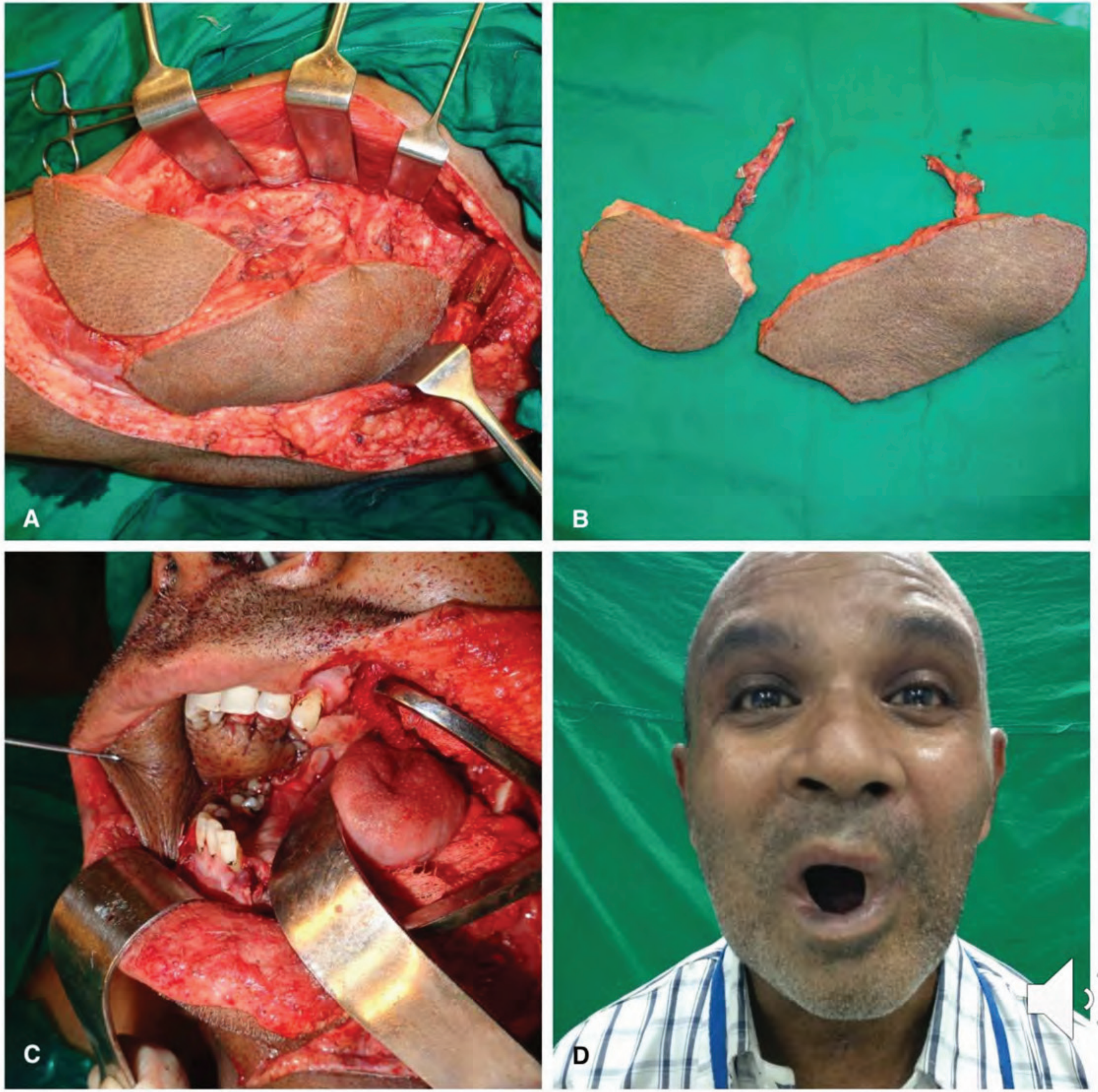

TFL flap

- TFL flap was first described by Hall and Nahai in 1978 *
- Extensively described as a transposition design or VY pedicle flap for trochanteric pressure sores
- Lateral thigh flap in breast reconstruction →

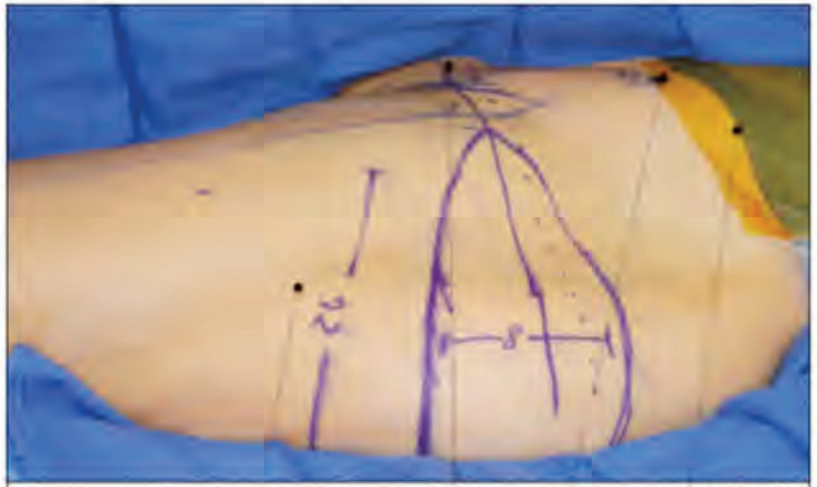

Image credit - Maricevich MA, Bykowski MR, Schusterman II MA, Katzel EB, Gimbel ML. Lateral thigh perforator flap for breast reconstruction: computed tomographic angiography analysis and clinical series. Journal of Plastic, Reconstructive & Aesthetic Surgery. 2017 May 1;70(5):577-84.

ALT - AMT

- Reciprocal relationship of the ALT and AMT perforators
- Salvage choice
  - Absence
  - Insufficiency or injury to ALT perforator
  - Chimeric flap with ALT.

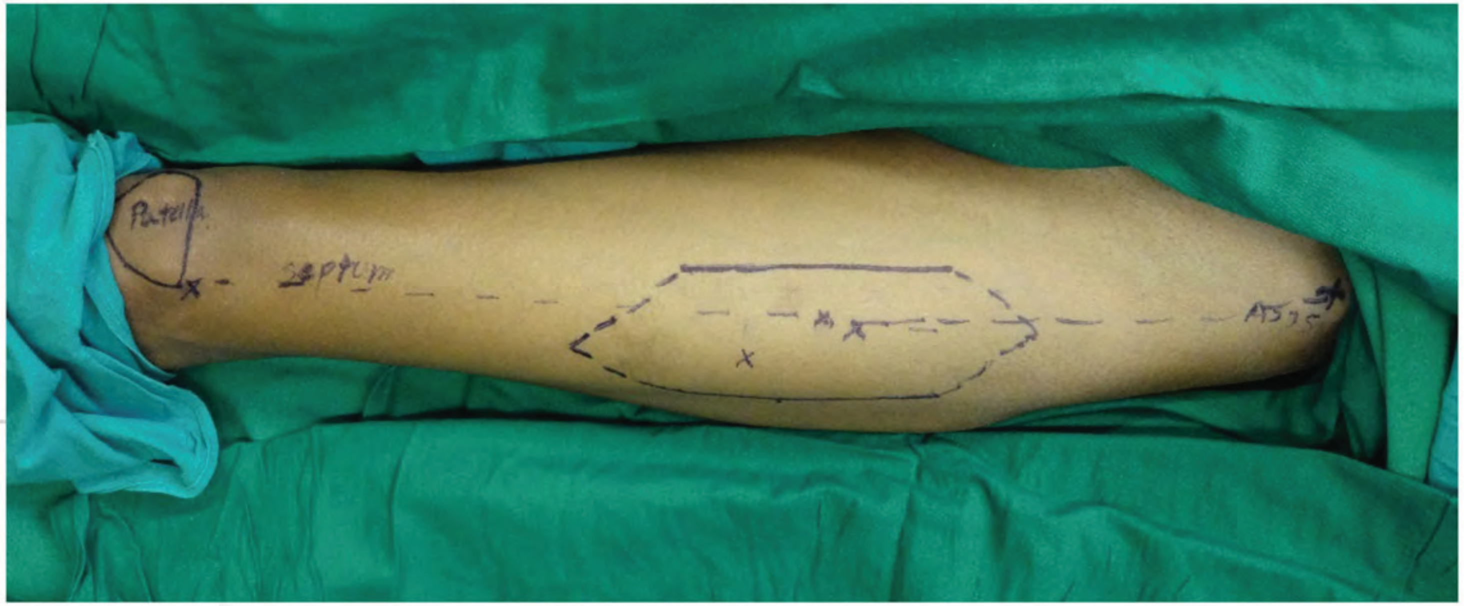

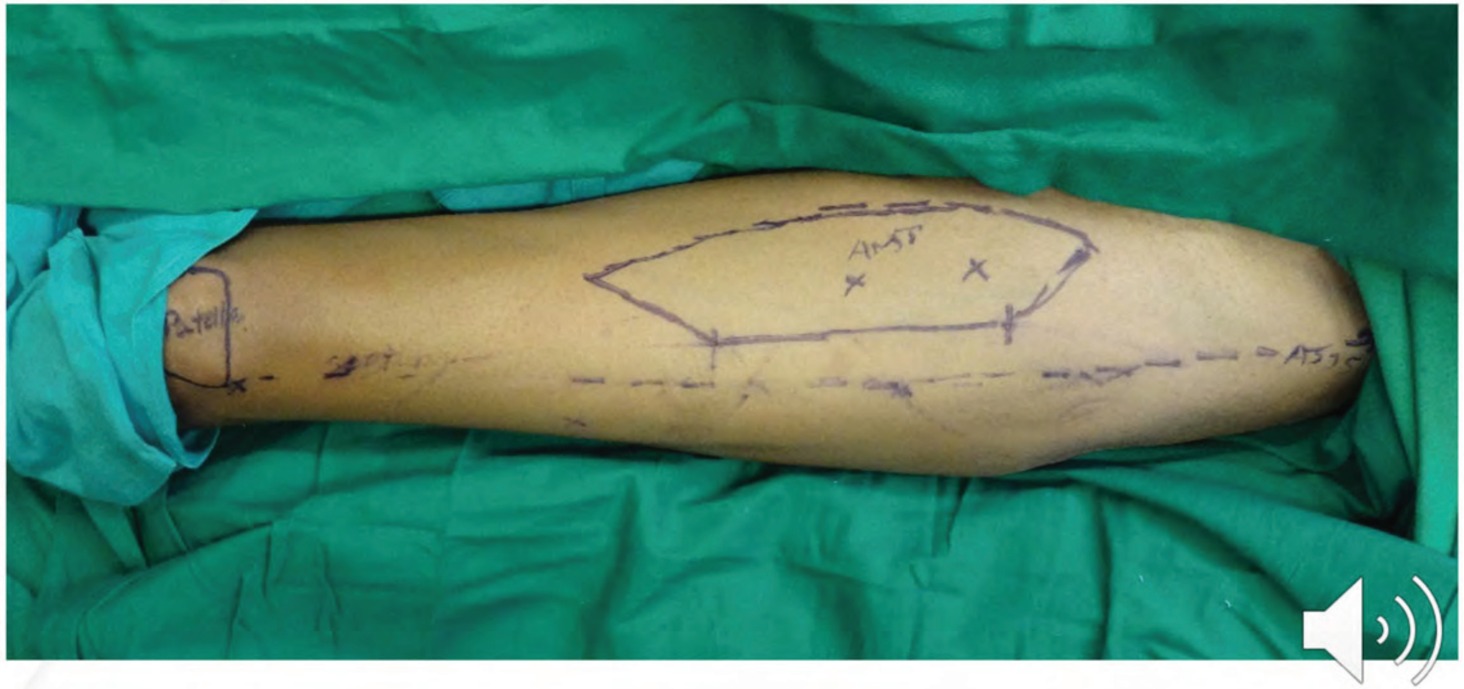

ALT – AMT - TFL

- Furthermore, complementary relationship exists between perforators in the ALT, AMT and TFL territory supplied by different tributaries of LFCA

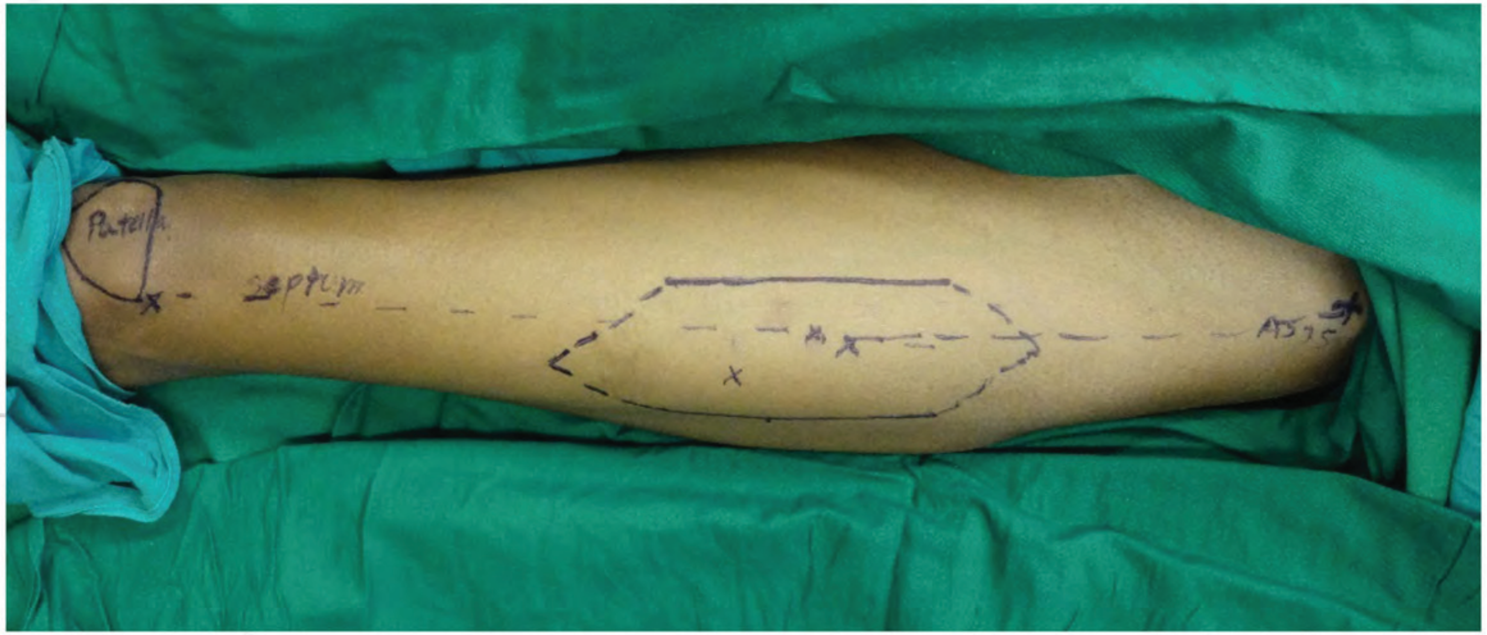

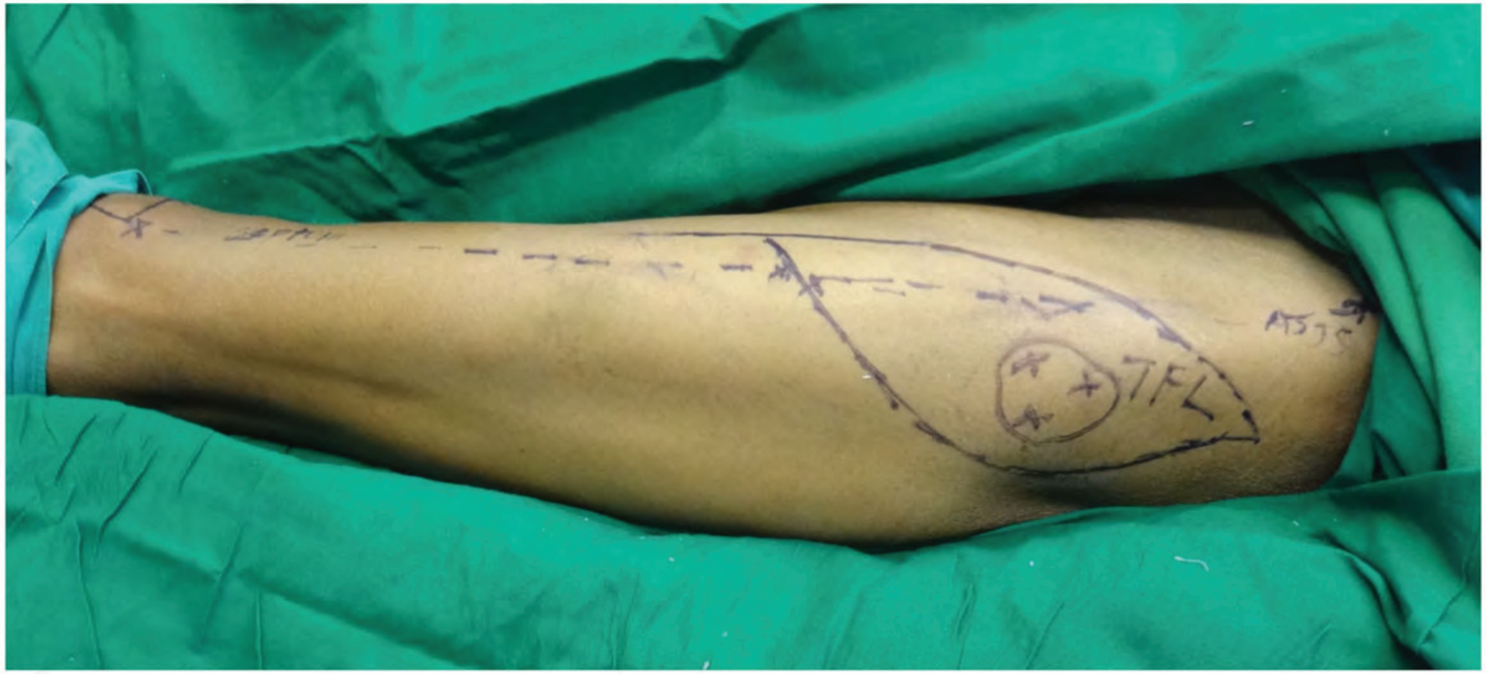

- Almost always a very large perforator, arising from the transverse branch of LCFA
- Pedicle length between 6-8cm

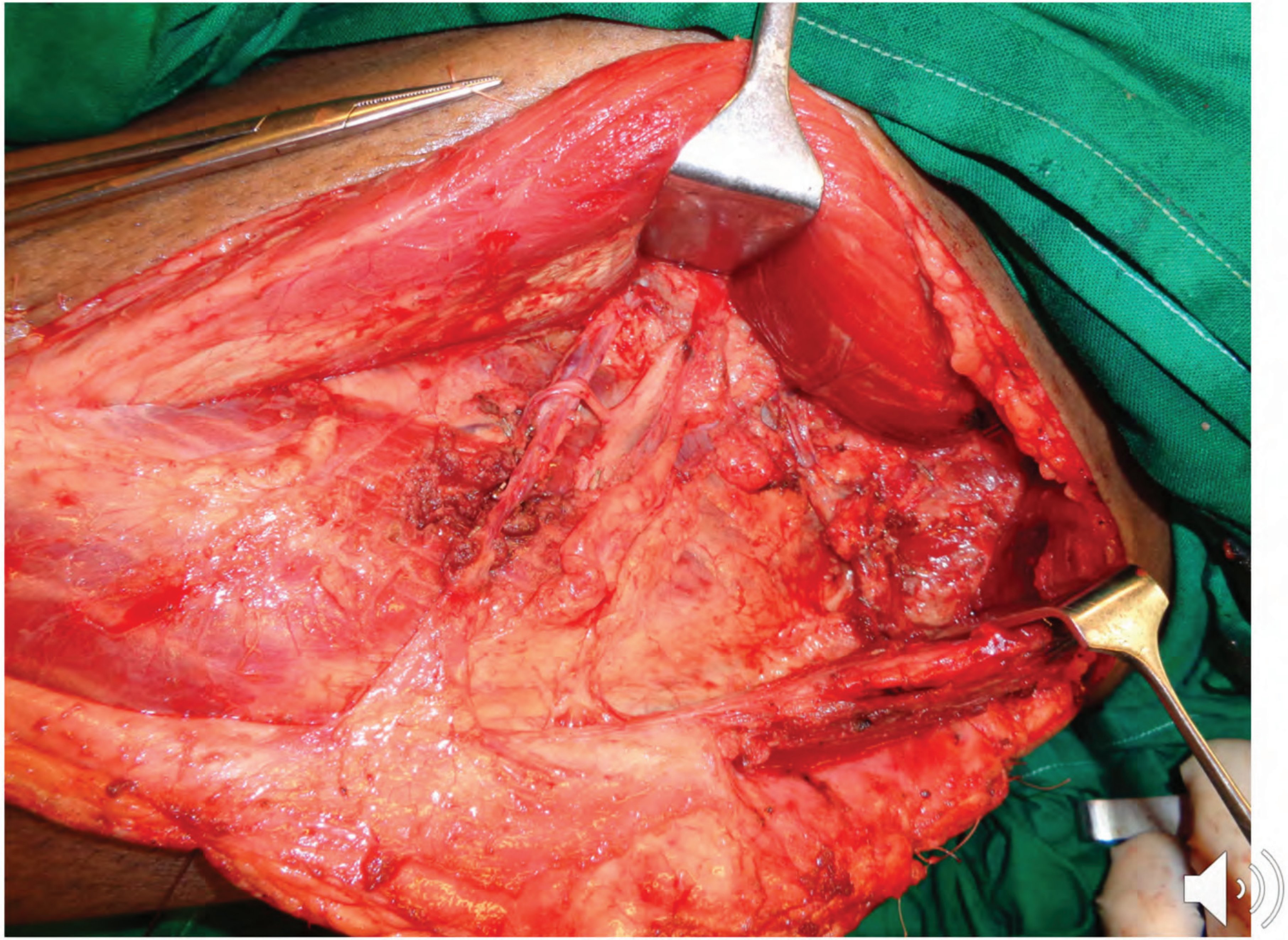

Perforator anatomy

- TFL enclosed between two fascial layers (Anterior and Posterior)

- Course of Perforators
  - Posterior Septo-cutaneous perforators (TFL and GM)
  - Musculo-cutaneous perforators
  - Anterior Septo-cutaneous perforators (TFL and RF/VL)

1. Sartorius
2. RF
3. TFL
4. GM

A. Anterior septum between TFL and RF/VL

B. Posterior Septum between TFL and GM

a. Septo-cutaneous perforators in anterior septum
b. Musculocutaneous perforators through TFL
c. Septo-cutaneous perforators in posterior septum

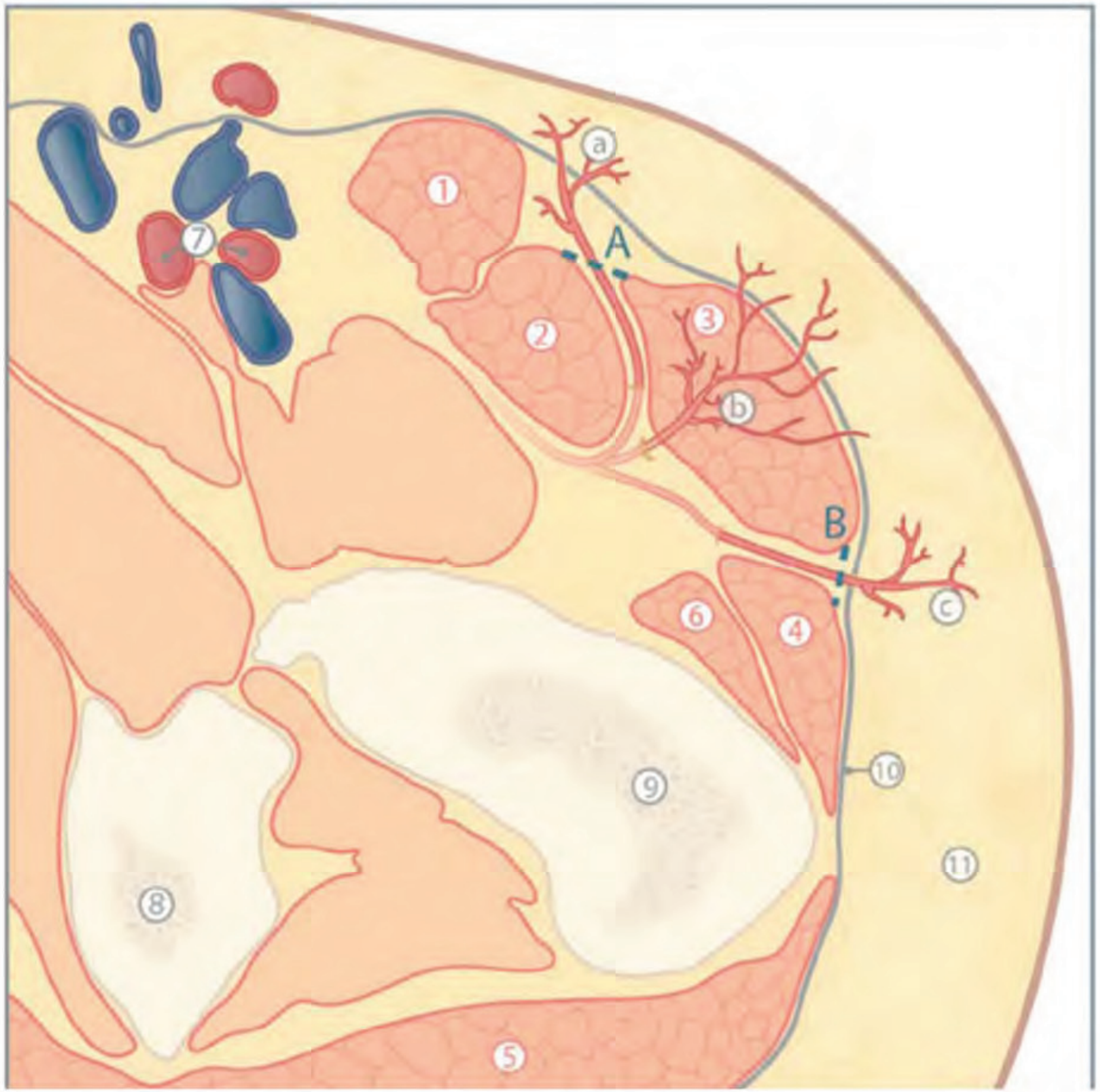

Image credit - Tuinder S, Lataster A, van der Hulst R. Lateral thigh perforator flap (septocutaneous tensor fasciae latae perforator flap). Perforator flaps for breast reconstruction. 2016(1):114-21.

Limitations

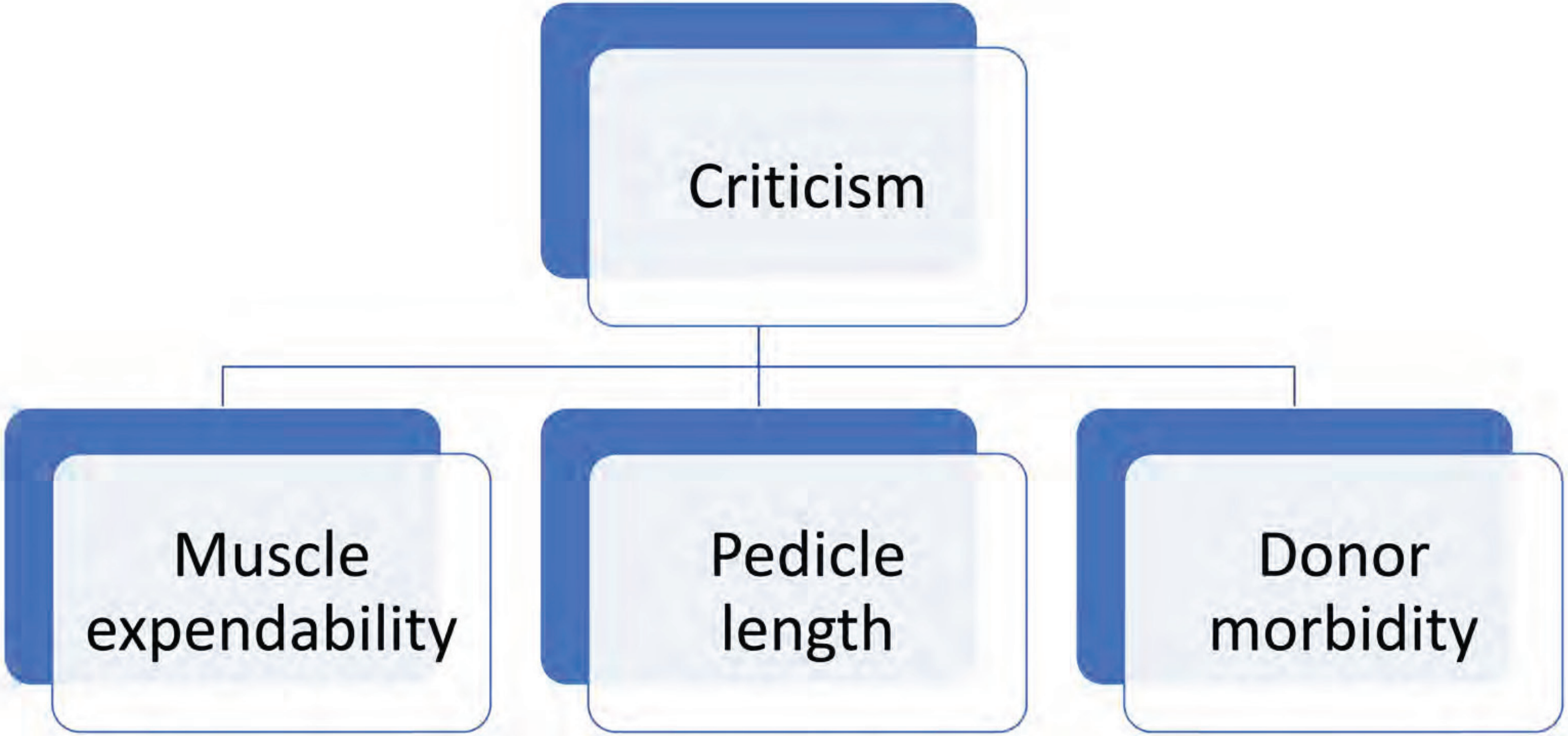

Muscle expendability

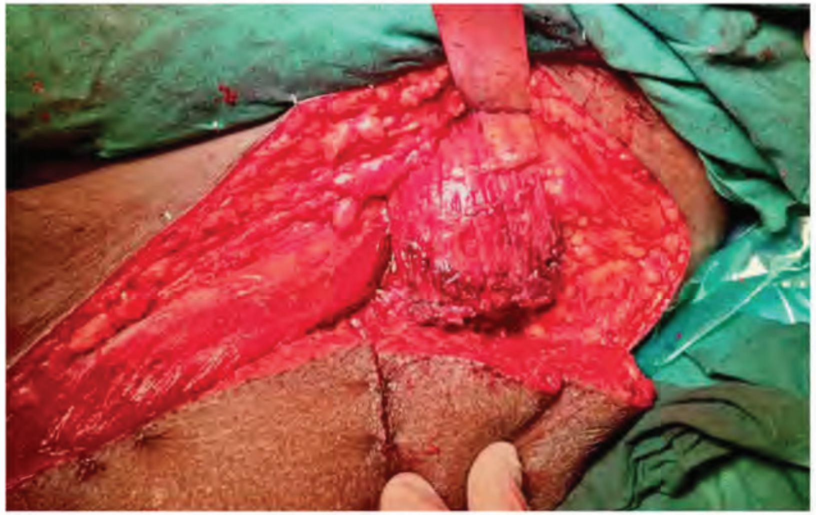

- TFL is a Type 1 muscle
- ***Usually,*** the muscle appeared ischemic and has to be removed.
- TFL as a muscle is not entirely expendable
- However, even with large ALT harvest → large deep fascia renders Fascia Lata functionless as it loses its insertion.

Short Pedicle Length

- In our Study – 6 to 8 cm
- All 29 patients had primary MVA
- No use of vein graft
- Just adequate length

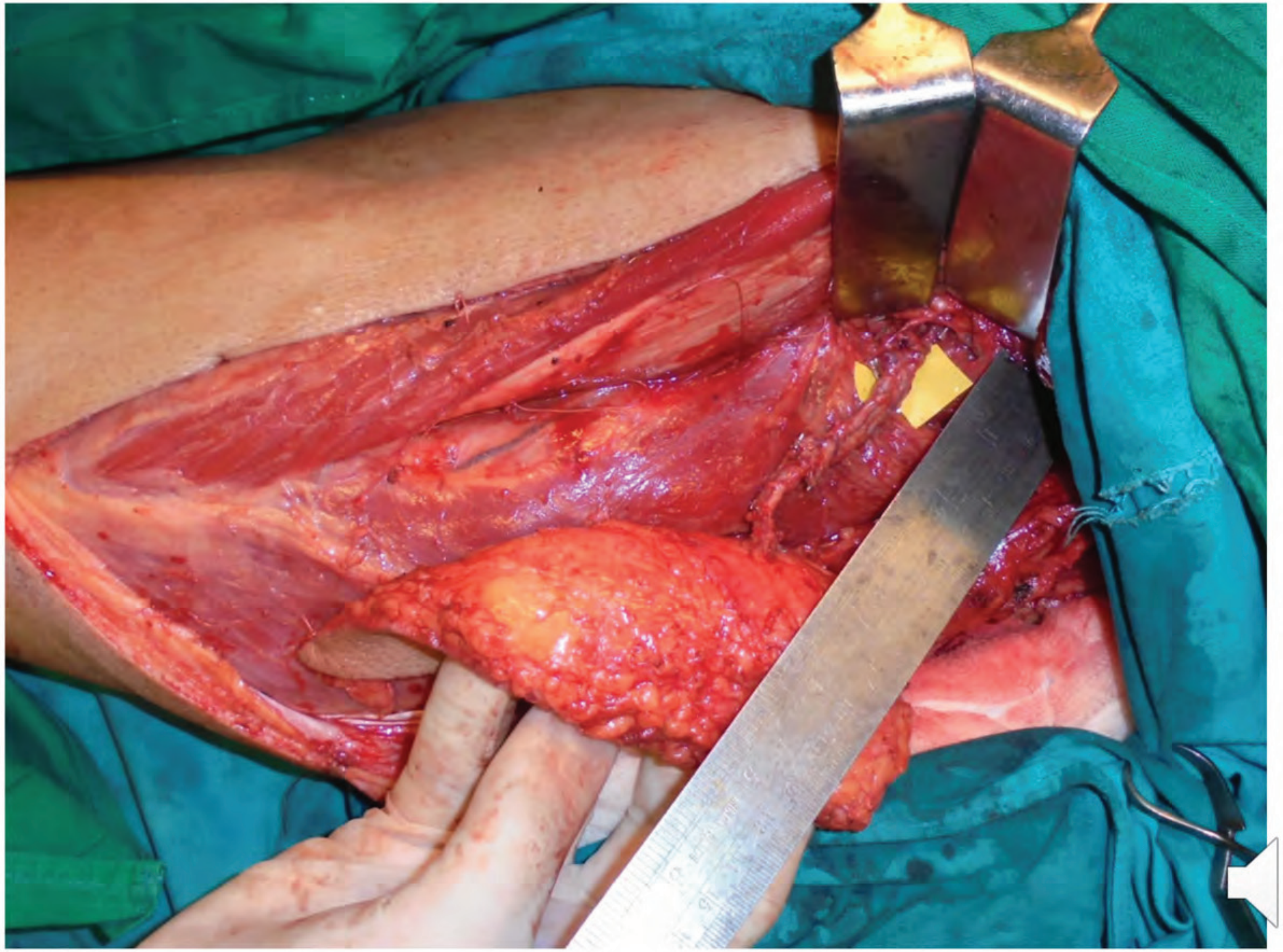

Donor

- Over greater trochanter → referable closed primarily without tension

- Closures under tension tends to break down.

- Not very conducive for primary skin grafts due to uneven surface or ischemic TFL muscle

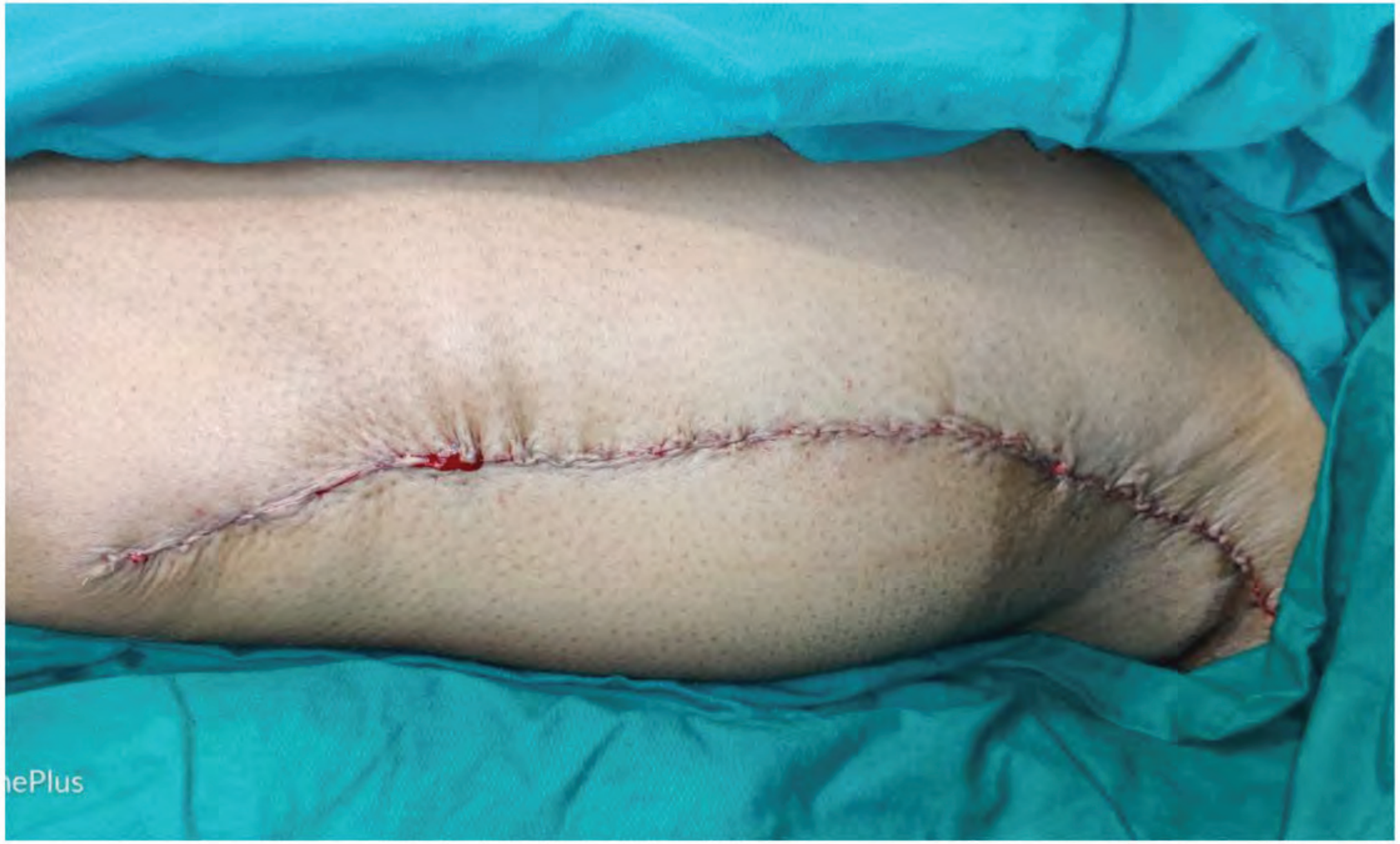

Conclusion

- Considering ALT, AMT and TFL as a complementary unit
- Consistent TFL septo-cutaneous perforator between the TFL and GM muscles
- Primary choice over ALT/AMT when small but thick flap is desired, especially in low BMI patients
- Conjoint, chimeric or multiple flaps with ALT more consistently and predictably

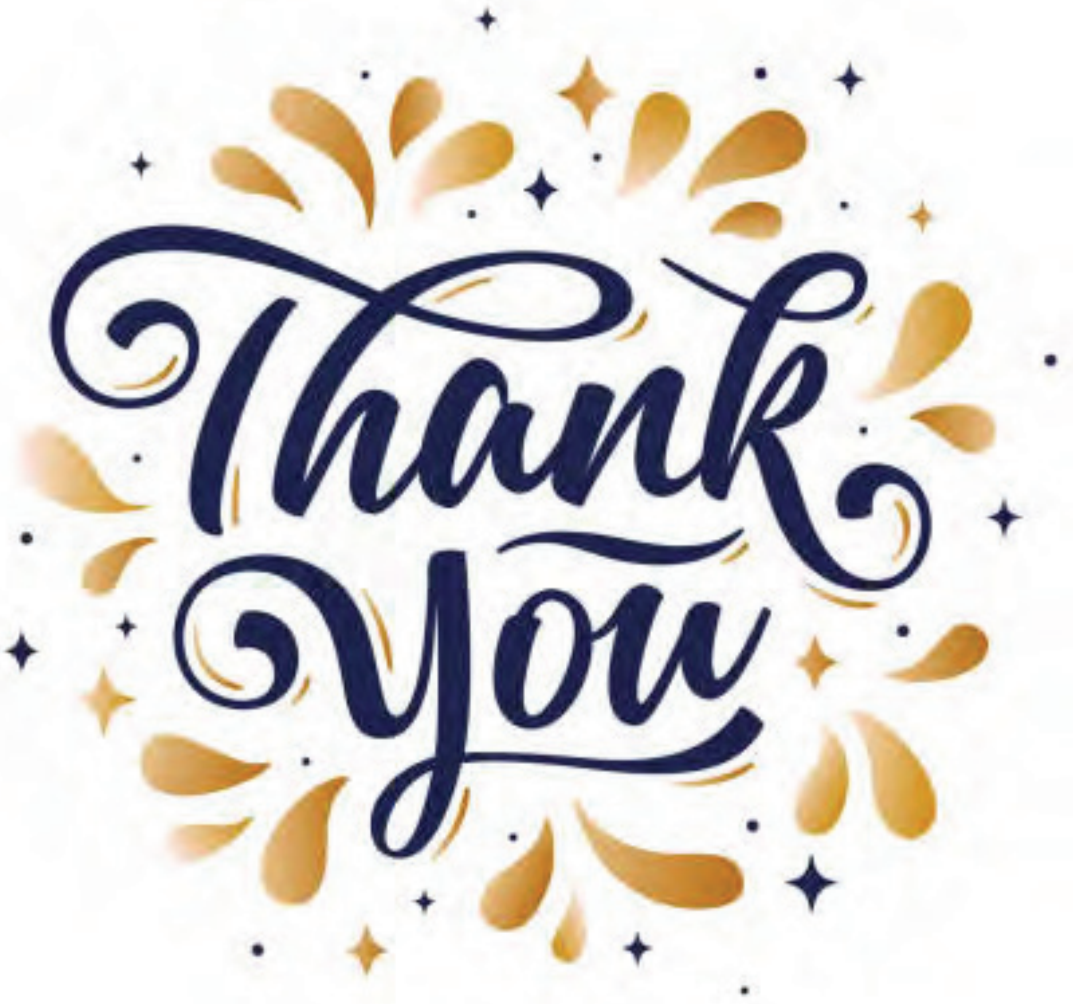
Thank You
